# New 6′-Amino-5′-cyano-2-oxo-1,2-dihydro-1′*H*-spiro[indole-3,4′-pyridine]-3′-carboxamides: Synthesis, Reactions, Molecular Docking Studies and Biological Activity

**DOI:** 10.3390/molecules28073161

**Published:** 2023-04-02

**Authors:** Victor V. Dotsenko, Nawras T. Jassim, Azamat Z. Temerdashev, Zainab R. Abdul-Hussein, Nicolai A. Aksenov, Inna V. Aksenova

**Affiliations:** 1 Department of Organic Chemistry and Technologies, Kuban State University, 149 Stavropolskaya St., 350040 Krasnodar, Russia; 2 Department of Chemistry, North Caucasus Federal University, 1a Pushkin St., 355017 Stavropol, Russia; 3 Department of Analytical Chemistry, Kuban State University, 149 Stavropolskaya St., 350040 Krasnodar, Russia; 4 Department of Pathological Analyses, College of Science, University of Basra, P.O. Box 49, Basrah 61004, Iraq

**Keywords:** isatins, malononitrile, monothiomalonamide, Michael addition, spiro-[indoline-3,4′-pyridines], docking studies, herbicide safeners

## Abstract

The purpose of this work was to prepare new isatin- and monothiomalondiamide-based indole derivatives, as well as to study the properties of the new compounds. The four-component reaction of 5-R-isatins (R = H, CH_3_), malononitrile, monothiomalonamide (3-amino-3-thioxo- propanamide) and triethylamine in hot EtOH yields a mixture of isomeric triethylammonium 6′-amino-3′-(aminocarbonyl)-5′-cyano-2-oxo-1,2-dihydro-1′*H*- and 6′-amino-3′-(aminocarbonyl)- 5′-cyano-2-oxo-1,2-dihydro-3′*H*-spiro[indole-3,4′-pyridine]-2′-thiolates. The reactivity and structure of the products was studied. We found that oxidation of spiro[indole-3,4′-pyridine]-2′-thiolates with DMSO-HCl system produced only acidification products, diastereomeric 6′-amino-5′-cyano-5-methyl-2-oxo-2′-thioxo-1,2,2′,3′-tetrahydro-1′H-spiro-[indole-3,4′-pyridine]- 3′-carboxamides, instead of the expected isothiazolopyridines. The alkylation of the prepared spiro[indole-3,4′-pyridine]-2′-thiolates upon treatment with N-aryl α-chloroacetamides and α-bromoacetophenones proceeds in a regioselective way at the sulfur atom. In the case of α-bromoacetophenones, ring-chain tautomerism was observed for the S-alkylation products. According to NMR data, the compounds consist of a mixture of stereoisomers of 2′-amino-6′-[(2-aryl-2-oxoethyl)thio]-3′-cyano-2-oxo-1′H-spiro[indoline-3,4′-pyridine]-5′-carboxamides and 5′-amino-3′-aryl-6′-cyano-3′-hydroxy-2-oxo-2′,3′-dihydrospiro[indoline-3,7′-thiazolo[3,2-a]pyridine]-8′-carboxamides in various ratios. The structure of the synthesized compounds was confirmed by IR spectroscopy, HRMS, ^1^H and ^13^C DEPTQ NMR studies and the results of 2D NMR experiments (^1^H-^13^C HSQC, ^1^H-^13^C HMBC). Molecular docking studies were performed to investigate suitable binding modes of some new compounds with respect to the transcriptional regulator protein PqsR of *Pseudomonas aeruginosa*. The docking studies revealed that the compounds have affinity for the bacterial regulator protein PqsR of *Pseudomonas aeruginosa* with a binding energy in the range of −5.8 to −8.2 kcal/mol. In addition, one of the new compounds, 2′-amino-3′-cyano-5-methyl-2-oxo-6′-{[2-oxo-2-(p-tolylamino)ethyl]thio}-1′H-spiro-[indoline-3,4′-pyridine]-5′-carboxamide, showed in vitro moderate antibacterial effect against *Pseudomonas aeruginosa* and good antioxidant properties in a test with 1,1-diphenyl-2-picrylhydrazyl radical. Finally, three of the new compounds were recognized as moderately active herbicide safeners with respect to herbicide 2,4-D in the laboratory experiments on sunflower seedlings.

## 1. Introduction

**Scheme 1:**
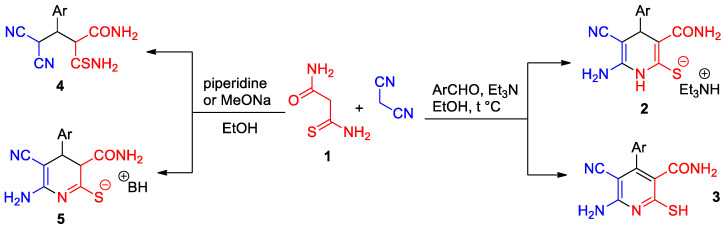
Possible pathways for reaction of monothiomalonamide **1** with malonononitrile and aldehydes.

Nicotinonitriles, nicotinamides and their partially saturated analogs represent a promising class of heterocyclic compounds with an interesting profile of biological activity (for reviews, see [1,2,3,4,5,6,7,8,9,10,11]). However, while nicotinonitriles have been fairly well studied, the related synthetic nicotinamides and 1,4-dihydronicotinamides have been less studied and require further investigation. One of the most accessible and efficient approaches to the synthesis of functionalized nicotinamides is based on the reaction of active methylene malonamides and malonthioamides with 1,3-C_3_ dielectrophiles [12,13,14,15,16,17,18,19,20,21,22]. Monothiomalonamide **1** (3-amino-3-thioxopropanamide) is known as a very convenient reagent for the preparation of substituted nicotinamides. It can be easily synthesized by passing H_2_S through a hot saturated solution of cyanoacetamide in pyridine in the presence of Et_3_N [23,24]. Substituted nicotinamides can be prepared by the reaction of monothiomalonamide **1** with malononitrile and aldehydes (or with arylmethylene malononitriles originated from Knoevenagel-type condensation) (Scheme 1). At the same time, it was noted that the structure of the reaction products strongly depends on the conditions. Thus, the paper [25] describes the preparation of 1,4-dihydropyridines **2** by reaction of monothiomalonamide **1** with aldehydes and malononitrile in the presence of Et_3_N in hot EtOH solution. However, other authors demonstrated the formation of nicotinamides **3** under similar reaction conditions [26]. As shown in paper [27], the reaction of thioamide **1** with benzylidene malononitrile may result in the formation of three different products, depending on the temperature, base and solvent used: the Michael adducts **4**, 1,4-dihydropyridin-2-thiolates similar to compounds **2**, or their 3,4-dihydro isomers **5** (Scheme 1). The formation of 3,4-dihydronicotinamides was also reported in other papers [13,21,28].

As highly reactive indole derivatives, isatins are very widely used in fine organic synthesis (for a review of isatin chemistry, see [29,30,31,32,33,34,35,36,37,38,39,40,41,42,43,44]). Isatin is also probably one of the most important compounds among the biologically active indoles. In recent years, isatin and isatin-based molecular hybrids have found a variety of applications in pharmacy and drug design [45,46,47,48,49,50,51,52,53,54,55,56,57,58,59,60,61,62].

Isatins have not previously been reacted with monothiomalonamide **1** and malononitrile. The expected products of spiro[indoline-3,4′-pyridine] structure are of interest as promising biologically active agents.

**Scheme 2:**
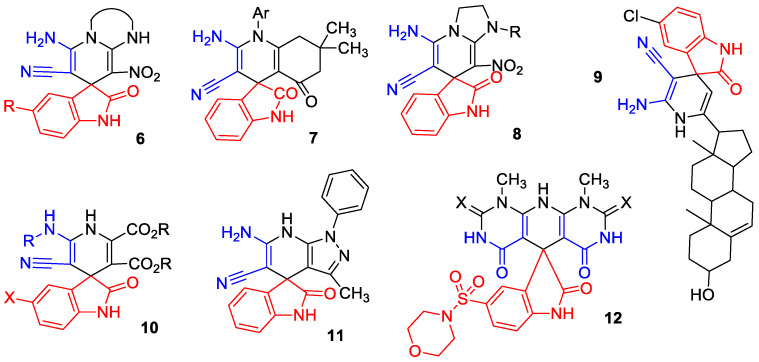
Biologically important spiro[indoline-3,4′-pyridines].

In particular, among the compounds with such structural motifs, acetyl and butyryl cholinesterase inhibitors **6** [63], antimicrobial agents **7** [64], insecticides **8** [65], antitumor agents **9** [66], on-off fluorescent chemosensors **10** for Cu^2+^ imaging in human hepatocellular liver carcinoma cells [67], fungicides **11 [68]** and SARS-CoV-2 inhibitors **12 [69]** were found (Scheme 2).

In the context of our interest in exploring the chemistry of biologically active nicotinonitriles [70,71,72,73,74,75,76,77,78,79] and indole–nicotinonitrile hybrids [80], it seemed reasonable to prepare new 6′-amino-5′-cyano-2-oxo-1,2-dihydro-1′*H*-spiro[indole-3,4′-pyridine]- 3′-carboxamides and to study their reactions and properties.

This work presents our research results concerning the reaction of monothiomalonamide **1** with isatins and malononitrile under basic conditions, as well as reactivity features, molecular docking studies and biological activities of the prepared 2′-amino-3′-cyano-2-oxospiro[indoline-3,4′-pyridine]-5′-carboxamides.

## 2. Results and Discussion

### 2.1. Synthesis

**Scheme 3:**
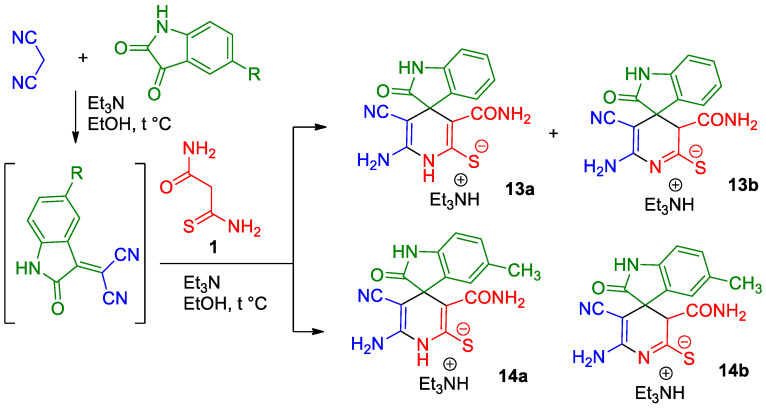
The reaction of isatins with malononitrile and monothiomalonamide **1** (3-amino-3-thioxopropanamide) in the presence of Et_3_N.

When a mixture of isatin and malononitrile was treated with excessive triethylamine and monothiomalonamide **1** (1.0 eq.), a white solid of a heterocyclization product was isolated in 88% yield. Detailed analysis of IR, ^1^H NMR, ^13^C DEPTQ NMR and ^1^H-^13^C HSQC and ^1^H-^13^C HMBC spectra revealed that the product was a mixture of triethylammonium 6′-amino-3′-(aminocarbonyl)-5′-cyano-2-oxo-1,2-dihydro-1′*H*-spiro-[indole-3,4′-pyridine]-2′-thiolate **13a** and 3′*H* isomeric thiolate **13b** in a molar ratio of ~7:1 (Scheme 3). A similar reaction with 5-methylisatin led to the formation of a mixture of 1′*H*-thiolate **14a** and 3′*H*-thiolate **14b** in a molar ratio of 7:3 and yield of 83%.

Full signal assignment for **13a** + **13b** and **14a** + **14b** mixtures was based on an analysis of the IR and NMR spectroscopy data, including ^13^C DEPTQ and 2D ^1^H-^13^C HSQC and ^1^H-^13^C HMBC experiments (Supplementary Data File). Thus, the ^1^H NMR spectra of **13a** + **13b** and **14a** + **14b** revealed a double set of aromatic proton signals and two peaks of NH_2_ protons (for major 1′H isomer at δ 5.36–5.37 ppm and for minor 3′H isomer at δ 6.23–6.25 ppm), as well as a common signal for NH isatin protons (δ 9.44–9.55 ppm) and one peak of pyridine NH proton of the major 1′H isomer at δ 7.67–7.69 ppm.

**Scheme 4:**
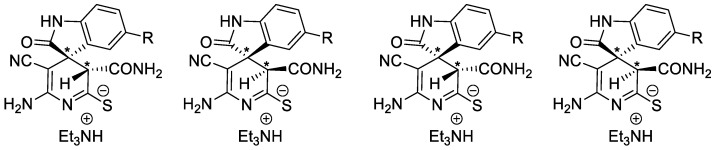
Diastereomeric pairs of minor isomers—2′-amino-5′-carbamoyl-3′-cyano- 2-oxo-3′H-spiro[indoline-3,4′-pyridine]-2′-thiolates **13b**,**14b**. An asterisk (*) marks the chiral centers in the molecules.

**Scheme 5:**
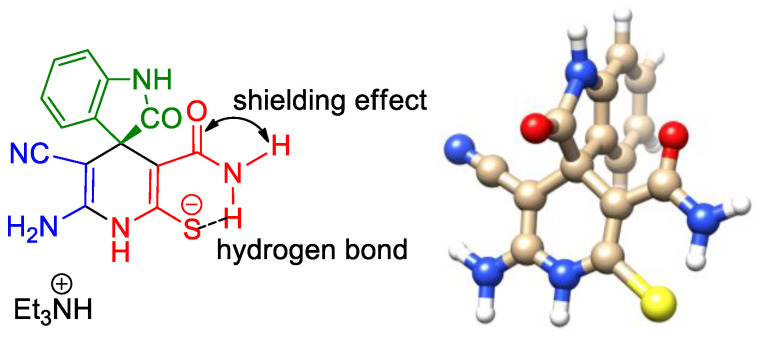
The local shielding/deshielding effects on the CONH_2_ protons.

Overall, the NMR spectra of the compounds **13** and **14** show a complex picture. It is noteworthy that in some cases the signals of the minor 3′H-isomers **13b**,**14b** exhibit signal doubling, apparently due to the splitting of the signals of two diastereomeric pairs (Scheme 4):

Noteworthy is the fact that the signals of the CONH_2_ protons were observed as two peaks with a very significant difference in chemical shifts (∆δ ≥ 4 ppm). Thus, for the major 1′H isomer, CONH_2_ protons appeared at δ 5.75–5.77 ppm and δ 10.25–10.26 ppm, while the peaks of the minor 3′H isomer were observed at δ 6.32–6.37 ppm and δ 10.19–10.30 ppm. We assume that the S-C=C-C(O)NH_2_ fragment is planar because of conjugation. The observed difference in chemical shifts can be explained by the presence of an intramolecular hydrogen bond between one of the CONH_2_ protons and the negatively charged sulfur atom on the one hand, and the anisotropic shielding effect of the carbonyl group on the second proton on the other (Scheme 5).

**Scheme 6:**
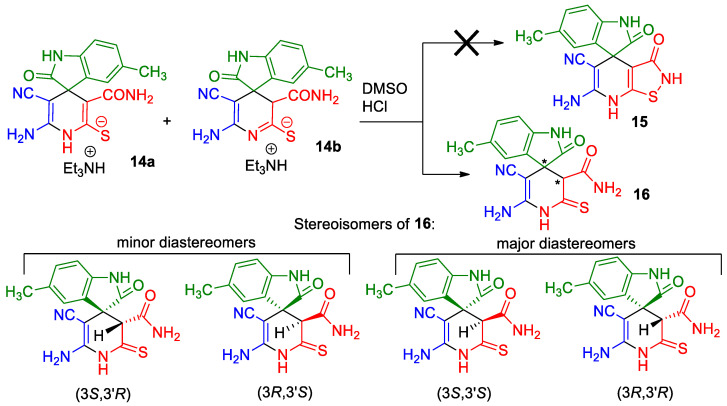
Synthesis and stereochemical features of 2-thioxopyridine **16**. An asterisk (*) marks the chiral centers in the molecules.

Due to the presence of a number of active functional groups, the resulting thiolates **13** and **14** seem to be attractive molecules for further transformations. Thus, in order to explore the reactivity of spiro[indole-3,4′-pyridine]-2′-thiolates, we attempted to oxidize thiolates **13** and **14** with dimethyl sulfoxide (DMSO)–HCl system. The oxidation of 2-mercaptonicotinamides and related compounds was reported as a general method for the synthesis of 3-oxo-2,3-dihydroisothiazolo[5,4-b]pyridines [24,81,82,83]. Such compounds are direct structural analogues of the practically important thieno[2,3-b]pyridines [84]. However, isothiazolo[5,4-b]pyridines are much less studied, although some compounds showed anti-tuberculosis [85] effects, were reported as COX-1 cyclooxygenase inhibitors [86], analgesics [87] and strong inhibitors of histone acetyltransferases with potential anticancer effects [88]. Previously, we have reported that the DMSO–HCl system can be successfully used as a mild oxidizing agent to convert 2-mercaptopyridin-3-carboxamides into isothiazolo[5,4-b]pyridines [72,89]. Nevertheless, upon treatment of thiolates **14a** + **14b** with excess HCl in DMSO solution, only corresponding 2-thioxopyridine **16** was isolated in 88% yield instead of the expected products **15.** According to NMR spectroscopy data, compound **16** exists in the solution as a mixture of diastereomers in the ratio ~3:5 (Scheme 6).

In actuality, there are two chiral centers in the molecule **16**; therefore, the compound can exist as a mixture of two diastereomeric pairs—(*3S,3′R*)/(*3R,3′S*) and (*3S,3′S*)(*3R,3′R*) (Scheme 6). The H-3′ proton signal of the minor isomers (δ 4.06 ppm) is noticeably shifted downfield with respect to the H-3′ signal of the major isomers (δ 3.78 ppm). Hence, we believe that the H-3′ proton of major isomer is affected by the anisotropic shielding effect of indoline C-2 carbonyl group, while the minor isomer H-3′ proton is not. Therefore, the (*3S,3′S*)/(*3R,3′R*) configuration should be assigned to the major diastereomers, and the (*3S,3′R*)(*3R,3′S*) configuration to the minor diastereomeric pair.

Alkylation of thiolates is a well-known approach to the diversity of heterocyclic thio ethers. Therefore, we decided to study the alkylation of thiolates **13** and **14** with some active α-halo carbonyls. It is well known that the alkylation of pyridine-2-thiolates and related compounds proceed in a regiospecific way at the sulfur atom. The S-alkylation products have been proven to be multifunctional reagents for fine organic synthesis and were recognized as biologically active compounds (for reviews, see [1,6,9,10,11,90,91,92]).

**Scheme 7:**
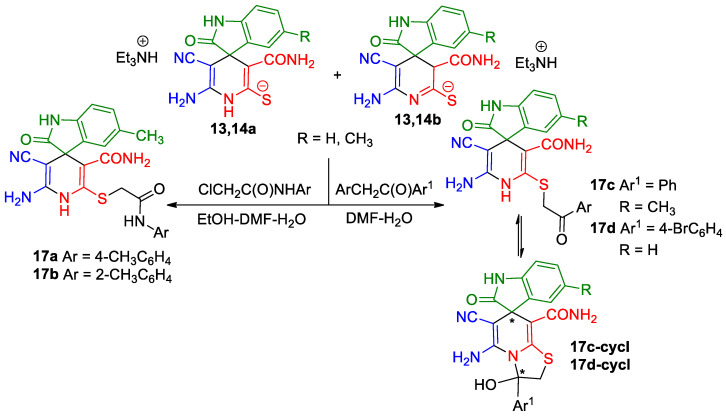
Alkylation of thiolates **13** and **14**. An asterisk (*) marks the chiral centers in the molecules.

As expected, upon treatment with N-aryl α-chloroacetamides and α-bromoacetophenones, thiolates **13** and **14** underwent regiospecific alkylation to give exclusively S-alkylation products in high yields. The structure of the products was confirmed by means of IR, ^1^H NMR, ^13^C NMR DEPTQ and HRMS spectroscopy. Analysis of spectral data of compounds **17** (Scheme 7) showed that the relevant products had the structure of 1′H-isomers only, while 3′H-isomers were not detected. This can be explained by the ease of prototropic migration 3′H→1′H with the formation of thermodynamically more stable products; a similar phenomenon was previously observed in some other S-alkylation reactions [13,28].

**Scheme 8:**
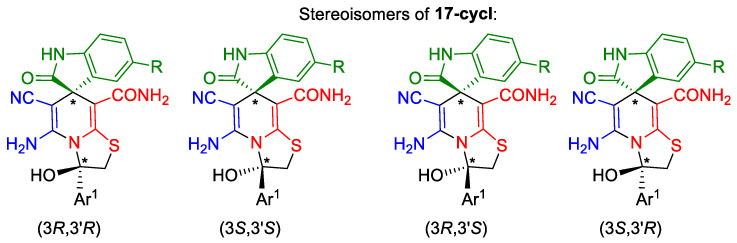
Diastereomeric pairs of thiazolo[3,2-a]pyridine tautomers of compounds **17c,d-cycl**. An asterisk (*) marks the chiral centers in the molecules.

It should also be noted that the spectra of carboxamides **17a,b** revealed the expected patterns, whereas the spectral picture of the alkylation products with α-bromacetophenones was very complex and contained signals of at least three different compounds. This pattern can be explained by the ring tautomerism of compounds **17c,d** in a solution. In addition, cyclic tautomers—thiazolo[3,2-a]pyridines **17c-cycl** and **17d-cycl** each have two stereogenic centers and are mixtures of two diastereomeric pairs (Scheme 8). It is noteworthy that similar ring-chain tautomerism was also previously observed in a number of related compounds [28,93].

The signals in the ^1^H NMR spectra of ring and chain forms of **17c,d** were assigned based on the integral intensities of SCH_2_ signals as well as on the presence of OH and pyridine NH proton signals. The major cyclic tautomers, thiazolo[3,2-a]pyridines **17c,d-cycl**, have two sets of two doublets shifted upfield (δ 3.18–3.26 and 3.54–3.58 ppm) with respect to the characteristic *AB*-quartet of SCH_2_C(O)Ar protons of acyclic minor forms **17c,d** (δ 4.71–4.76 ppm, ^2^*J* = 17.2 Hz) (Supplementary Data File).

Nevertheless, the unambiguous assignment of signals in the NMR spectra of all tautomers and stereoisomers in the **17c,d** system seems difficult. Additionally, the question of which tautomeric form of **17c,d** is preferred in the solid state remains unclear; the presence of a strong absorption band at ν 1697–1701 cm^−1^, which may be assigned to keto carbonyls, and the absence of band doubling indirectly indicate the preference of noncyclic forms. However, other authors [93] showed, using an X-ray method, that compounds with such type of ring-chain tautomerism in the solid state exist as thiazolo[3,2-a]pyridines. In general, this question requires further exploration.

### 2.2. Docking Studies

First, we used the Osiris property explorer on-line tool [94] for in silico prediction of the drug likeness of the synthesized compounds. Salts **13** and **14**, as well as tautomeric mixtures **17c,d**, were excluded from further consideration. All the molecules **16,17a,b** were found to be non-toxic in terms of mutagenicity, carcinogenicity, skin irritancy and reproductive effects.

Many indoles/isatins, nicotinonitriles and hybrid compounds combining fragments of the above systems are of interest as antimicrobial agents. Recently, indole-containing compounds have been reported to have antibacterial activity as well as activity as resistance-modifying agents [95,96,97,98]. It is noteworthy that infections caused by *Pseudomonas aeruginosa* pose a real problem, especially in critically ill and immunodeficient patients. The main cause of high mortality is the emergence of drug-resistant strains [99].

For this reason, we decided to investigate the antibacterial potential of the new compounds **16,17a,b** against *Pseudomonas aeruginosa*. The LysR-type transcriptional regulator protein PqsR (PDB ID 4JVC) was chosen as a target. This bacterial transcriptional regulator protein is a key component of alkyl-quinolone-dependent quorum sensing in *Pseudomonas aeruginosa* and could be considered as a potential target for new antibacterial agents that attenuate infection by blocking virulence [100].

The energy-minimized 3D structures for each individual stereoisomer of ligand molecules **16,17a,b** were docked using Autodock Vina against the bacterial regulator protein PqsR of *Pseudomonas aeruginosa* (PDB ID 4JVC) at standard precision mode (SP). The docking score of all the ligand molecules **16,17a,b** were found to be in the range of −5.8 to −8.2 kcal/mol.

**Figure 1:**
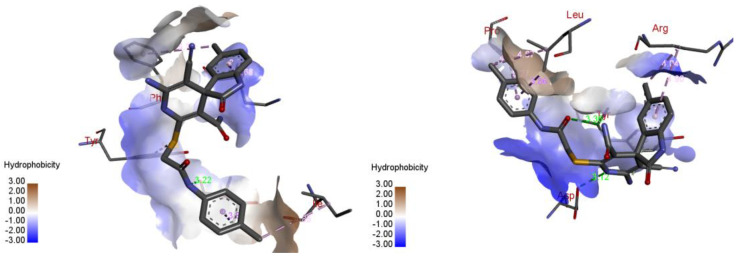
Best-scored docking poses for (*R*)-**17a** (**left**) and (*S*)-**17a** (**right**) with bacterial protein PqsR (PDB ID 4JVC) (dotted lines show non-valent contacts of the ligand with amino acid residues of the target protein PqsR).

**Figure 2:**
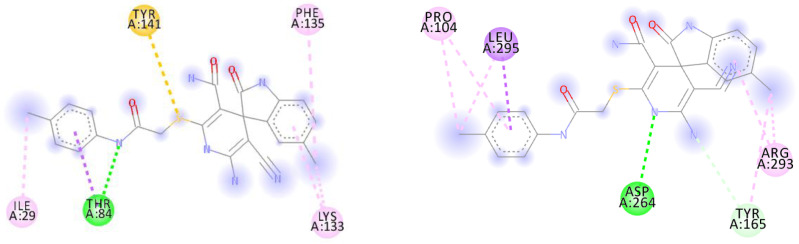
Two-dimensional docking pose interaction diagram with the key amino acids in bacterial protein PqsR binding site for (*R*)-**17a** (**left**) and (*S*)-**17a** (**right**) (conventional hydrogen bonds showed green, Pi-Sigma interactions showed purple, Pi-Sulfur interaction showed yellow and alkyl and Pi-alkyl interactions showed pink).

The docking results are presented in Table 1. As follows from Table 1, both the (*R*)- and (*S*)-enantiomers of compound **17a** had the minimum value of the scoring function and hence showed the best affinity to the protein target. The major interactions between the ligands with the target protein can be categorized as “hydrogen bonding, hydrophobic”, which was crucial to stabilize the inhibitors inside the binding pocket of the receptor. The most important interactions are shown in Figure 1 and Figure 2.

### 2.3. Antibacterial and Antioxidant Activity of Compound ***17a***

The leader compound **17a** was also tested in vitro to determine its ability to inhibit pathogen growth by the agar well-diffusion method using Mueller–Hinton agar at 100 mg/mL concentration. Antibacterial activity was tested against two strains of Gram-negative bacteria: *Escherichia coli ATCC 25922* and *Pseudomonas aeruginosa ATCC 27853*. The reference drug was Ciprofloxacin. The results of in vitro studies showed that **17a** efficiently suppresses Gram-negative bacteria *P. aeruginosa ATCC 27853*, but has no effect against Gram-positive bacteria. Compound **17a** also demonstrated a moderate inhibitory effect against *E. coli*. Then, the minimum inhibitory concentration (MIC) value for **17a** was determined based on the results of the examination according to the protocol described in [101]. Ciprofloxacin was used as the reference drug. We found that compound **17a** showed moderate antibacterial activity with MIC 12.5 μg/mL (Ciprofloxacin—1 μg/mL).

The ability of **17a** to scavenge DPPH radical (1,1-diphenyl-2-picrylhydrazyl) was examined by the method proposed by Blois [102]. Antioxidant activity was calculated in % of inhibition according to Equation (1):

(1) $$I=\frac{A_{control}-A_{test}}{A_{control}}\times 100\%,$$

where *I*—inhibition effect, %; *A_control_*—absorption in control experiment; and *A_test_*—absorption in test with compound **17a**.

The results are shown in Table 2. As follows from Table 2, compound **17a** showed antioxidant effects comparable to those of ascorbic acid.

### 2.4. Agrochemical Studies

Some of the new compounds were tested as herbicide safeners with respect to 2,4-dichlorophenoxyacetic acid (2,4-D). 2,4-D is widely used and non-toxic for human herbicides [103]. However, 2,4-D is toxic for sunflowers. If the recommended dose for controlling weeds in resistant cereal crops is 0.5–0.8 kg/ha of 2,4-D by the active substance, the 15–18 g/ha dose for sunflower leads to a 40–60% decrease in yield.

One of the most effective approaches to increase plant resistance towards herbicides is the activation of metabolic processes affected by herbicide safeners (also called herbicide antidotes or detoxifiers) [104,105]. Herbicide safeners can be defined [106] as agrochemicals suitable for neutralization of phytotoxins in plants, thus protecting crop plants from herbicide injury. Safeners are harmless to crop plants (or even have a growth-stimulating effect), but do not affect the activity of herbicides against weeds.

It is known that some functionalized pyridines are efficient herbicide safeners and plant growth regulators [107,108,109,110,111]. Nicotinamides **16a** and **17a,b** were studied as 2,4-D antidotes with respect to sunflower seedlings using the reported procedure [107] (see the Materials and Methods). The antidote effect *A* was determined as a ratio of the hypocotyl (or root) length of sunflower seedlings in the “herbicide + antidote” experiments to the length in the reference group (where the seedlings were treated with 2,4-D only) (Equation (2)):

*A* = (*L*_exp_/*L*_ref_) × 100%, (2)

where *L*_exp_ is an organ length (mm) in the group of seedlings treated with 2,4-D and tested compound, and *L*_ref_ is an organ length (mm) in the reference group of sunflower seedlings. We found that compounds **16a** and **17a,b** showed moderate 2,4-D antidote effect in the laboratory experiments (Table 3). As we can see, spiro[indole-3,4′-pyridines] **16a** and **17a,b** reduced the negative effect of 2,4-D on sunflower seedling hypocotyls by 16–33% and by 12–25% on sunflower seedling roots.

## 3. Materials and Methods

^1^H and ^13^C DEPTQ NMR spectra and 2D NMR experiments were recorded in solutions of DMSO-d_6_ on a Bruker AVANCE-III HD instrument (at 400.40 or 100.61 MHz, respectively). Residual solvent signals were used as internal standards in DMSO-d_6_—2.49 ppm for ^1^H, and 39.50 ppm for ^13^C nuclei. HRMS spectra were recorded using a Bruker MaXis Impact quadrupole time-of-flight mass spectrometer equipped with an electrospray ionization source in positive ion detection mode. The voltage at the ionization source was 3.5 kV, the drying gas flow rate was 8 L/min, the spray gas pressure was 2 bar, the temperature of the ionization source was 250 °C, the mass scanning range (*m*/*z*) was 50–1000 and the scanning speed was 3 Hz. The data were processed using Bruker Data Analysis 4.1 software. See Supplementary Materials File for NMR, FTIR and HRMS spectral charts.

FT-IR spectra were measured on a Bruker Vertex 70 instrument equipped with an ATR sampling module. Elemental analyses were carried out using a Carlo Erba 1106 Elemental Analyzer. Reaction progress and purity of isolated compounds were controlled by TLC on Sorbfil-A plates, eluent—acetone:hexane 1:1 or ethyl acetate:light petroleum 3:1, the spots were visualized in UV-light and iodine vapors. Monothiomalonamide **1** (3-amino-3-thioxopropanamide) was prepared from α-cyanoacetamide and hydrogen sulfide as described earlier [23,24]. Isatins, triethylamine, malononitrile and solvents were purchased from commercial vendors.

**The reaction of isatins, malononitrile and monothiomalonamide**. In a 50 mL beaker, 0.5 g (7.57 mmol) of malononitrile, 7.57 mmol of the corresponding isatin (1.11 g of isatin or 1.22 g of 5-methylisatin) and 20 mL of EtOH were added. Two drops of triethylamine were added to the suspension under heating (50 °C) and stirring. The reaction mixture became dark red due to the formation of the corresponding Knoevenagel condensation product. Stirring at 50 °C was continued for another 0.5 h, after which 0.89 g (7.57 mmol) of monothiomalonamide **1** and 1.6 mL (11.35 mmol, 1.5 eq.) of triethylamine were added. The mixture was stirred under vigorous heating (50 °C) for 2 h, then cooled and kept in a freezer overnight. The precipitate was filtered off and washed with acetone until colorless washings were obtained and then dried at 60 °C.

*Triethylammonium 6′-amino-3′-(aminocarbonyl)-5′-cyano-2-oxo-1,2-dihydro-1′H-spiro-[indole-3,4′-pyridine]-2′-thiolate **13a** and isomeric 6′-amino-3′-(aminocarbonyl)-5′-cyano-2-oxo- 1,2-dihydro-3′H-spiro-[indole-3,4′-pyridine]-2′-thiolate **13b**.* Off-white solid, yield was 2.74 g (88%). FTIR, ν_max_, cm^−1^: 3395, 3323, 2710 (N–H, ^+^N–H); 2170 (C≡N); 1715, 1684 (C=O).

^1^H NMR (400 MHz, DMSO-*d*_6_): the signals of major isomer **13a**—1.10 (t, ^3^*J* = 7.2 Hz, 9H, 3 CH_3_CH_2_N), 2.90–2.95 (m, 6H, 3 CH_3_CH_2_N), 5.37 (br s, 2H, NH_2_), 5.77 (br s, 1H, C(O)NH_2_), 6.55 (d, ^3^*J* = 7.5 Hz, 1H, H-7 indole), 6.70–6.74 (m, 1H, H-5 indole), 6.78 (d, ^3^*J* = 7.0 Hz, 1H, H-4 indole), 6.89–6.93 (m, 1H, H-6 indole), 7.69 (s, 1H, NH pyridine), 9.55 (s, 1H, NH indole), 10.25 (br s, 1H, C(O)NH_2_); the observed signals of minor isomer **13b**—1.10 (t, ^3^*J* = 7.2 Hz, 9H, 3 CH_3_CH_2_N), 2.90–2.95 (m, 6H, 3 CH_3_CH_2_N), 3.80 (s, 1H, 3′H), 6.25 (br s, 2H, NH_2_), 6.34–6.37 (m, 1H, C(O)NH_2_), 6.75–6.76 (m, 1H, H-indole), 6.93–6.96 (m, 1H, H-indole), 7.10–7.14 (m, 1H, H-indole), 9.55 (s, 1H, NH indole), 10.30 (br s, 1H, C(O)NH_2_). NH^+^ signal was not observed, probably due to the fast H–D exchange. According to the integral signal intensities in the ^1^H NMR spectrum, the ratio of 1′H-isomer **13a** to 3′H-isomer **13b** is about 7:1.

^13^C DEPTQ NMR (101 MHz, DMSO-*d*_6_): the signals of major isomer **13a**—9.3* (NCH_2_CH_3_), 45.7 (NCH_2_CH_3_), 52.2 (C spiro), 56.5 (C-5′), 100.4 (C-3′), 107.8* (CH-7 indole), 120.2* (CH-5 indole), 121.1 (C≡N), 122.3* (CH-4 indole), 125.7* (CH-6 indole), 141.0 (C-3a indole), 141.5 (C-7a indole), 150.3 (C-6′), 159.1 (C-2′), 170.0 (CONH_2_), 182.2 (C=O); the observed signals of minor isomer **13b**—9.3* (NCH_2_CH_3_), 45.7 (NCH_2_CH_3_), 58.9* (CH-3′), 109.1* (CH-7 indole), 119.9 (C≡N), 121.8* (CH-5 indole), 124.2* (CH-4 indole), 128.1* (CH-6 indole), 135.3 (C-3a indole), 137.2 (C-7a indole), 151.8 (C-6′). *Negatively phased signals.

HRMS (ESI) m/z: calculated for C_20_H_27_N_6_O_2_S [M + H]^+^: 415.1916, found 415.1912 (Δ 1.01 ppm).

*Triethylammonium 6′-amino-3′-(aminocarbonyl)-5′-cyano-5-methyl-2-oxo-1,2-dihydro- 1′H-spiro[indole-3,4′-pyridine]-2′-thiolate **14a** and isomeric 6′-amino-3′-(aminocarbonyl)-5′- cyano-5-methyl-2-oxo-1,2-dihydro-3′H-spiro[indole-3,4′-pyridine]-2′-thiolate* ***14b***. Off-white solid, yield was 2.68 g (83%). FTIR, ν_max_, cm^−1^: 3406, 3377, 2711 (N–H, ^+^N–H); 2172 (C≡N); 1676, 1666 (C=O).

^1^H NMR (400 MHz, DMSO-*d*_6_): the signals of major isomer **14a**—1.11 (t, ^3^*J* = 7.2 Hz, 9H, 3 CH_3_CH_2_N), 2.17 (s, 3H, ArCH_3_), 2.93–2.98 (m, 6H, 3 CH_3_CH_2_N), 5.36 (br s, 2H, NH_2_), 5.75 (br s, 1H, C(O)NH_2_), 6.44 (d, ^3^*J* = 7.5 Hz, 1H, H-7 indole), 6.61 (br s, 1H, H-4 indole), 6.72 (d, ^3^*J* = 7.5 Hz, 1H, H-6 indole), 7.67 (s, 1H, NH pyridine), 9.44 (s, 1H, NH indole), 10.26 (br s, 1H, C(O)NH_2_); the observed signals of minor isomer **14b**—1.11 (t, ^3^*J* = 7.2 Hz, 9H, 3 CH_3_CH_2_N), 2.20 (s, 3H, ArCH_3_), 2.93–2.98 (m, 6H, 3 CH_3_CH_2_N), 4.35 (s, 1H, 3′H), 6.23 (br s, 2H, NH_2_), 6.32–6.35 (m, 1H, C(O)NH_2_), 6.64 (d, ^3^*J* = 7.7 Hz, 1H, H-7 indole), 6.76 (br s, 1H, H-4 indole), 6.93 (d, ^3^*J* = 7.7 Hz, 1H, H-6 indole), 9.44 (s, 1H, NH indole), 10.19 (br s, 1H, C(O)NH_2_); NH^+^ signal was not observed, probably due to the fast H–D exchange. According to the integral signal intensities in the ^1^H NMR spectrum, the ratio of 1′H-isomer **14a** to 3′H-isomer **14b** is about 7:3.

^13^C DEPTQ NMR (101 MHz, DMSO-*d*_6_): the signals of major isomer **14a**—9.2* (NCH_2_CH_3_), 20.8* (ArCH_3_), 45.7 (NCH_2_CH_3_), 52.2 (C spiro), 56.7 (C-5′), 100.5 (C-3′), 107.6* (CH-7 indole), 121.2 (C≡N), 123.1* (CH-4 indole), 126.0* (CH-6 indole), 128.6 (C-5 indole), 139.0 (C-7a indole), 141.1 (C-3a indole), 150.3 (C-6′), 159.0 (C-2′), 170.0 (CONH_2_), 182.2 (C=O); the observed signals of minor isomer **14b**—9.2* (NCH_2_CH_3_), 20.6* (ArCH_3_), 45.7 (NCH_2_CH_3_), 53.1* (CH-3′), 107.5 (C-5′), 108.9* (CH-7 indole), 120.0 (C≡N), 124.8* (CH-4 indole), 128.4* (CH-6 indole), 130.5 (C-5 indole), 135.4 (C-7a indole), 139.1 (C-3a indole), 151.7 (C-6′), 178.4 (C=O); *Negatively phased signals.

HRMS (ESI) *m/z*: calculated for C_21_H_29_N_6_O_2_S [M + H]^+^: 429.2073, found 429.2069 (Δ 0.86 ppm).

**Synthesis of 6′-amino-5′-cyano-5-methyl-2-oxo-2′-thioxo-1,2,2′,3′-tetrahydro-1′H- spiro[indole-3,4′-pyridine]-3′-carboxamide 16**. A 10 mL beaker was charged with 300 mg (0.7 mmol) of thiolate **14a** + **14b** and 2 mL of DMSO. To the solution formed, 0.2 mL of concentrated hydrochloric acid was added dropwise under vigorous stirring. Gas evolution (*Caution! Me_2_S!*) and formation of a precipitate were observed. The mixture was stirred for 10 min, diluted with EtOH up to a volume of 10 mL, filtered off, washed with EtOH and petroleum ether and dried at 60 °C. Compound **16** was obtained as a yellow finely crystalline powder, yield 201 mg (88%).

FTIR, ν_max_, cm^−1^: 3425, 3317, 3207 (N–H); 2193 (C≡N); 1705, 1653 (C=O).

^1^H NMR (400 MHz, DMSO-*d*_6_): the signals of major (*3S,3′S*)/(*3R,3′R*) diastereomers of **16**—2.22 (s, 3H, ArCH_3_), 3.78 (s, 1H, H-3′), 6.32 (br s, 2H, NH_2_), 6.72 (d, ^3^*J* = 7.8 Hz, 1H, H-7 indole), 6.90–6.97 (m, 1H, H-4 indole, overlapped with the signals CONH_2_ and Ar–H of minor diastereomers), 7.03 (d, ^3^*J* = 7.8 Hz, 1H, H-6 indole), 7.15 (br s, 1H, CONH_2_), 7.33 (br s, 1H, CONH_2_), 10.40 (s, 1H, NH indole), 11.59 (br s, 1H, C(S)NH); the signals of minor (*3S,3′R*)(*3R,3′S*) diastereomers of **16**—2.23 (s, 3H, ArCH_3_), 4.06 (s, 1H, H-3′), 6.35 (br s, 2H, NH_2_), 6.62 (d, ^3^*J* = 7.8 Hz, 1H, H-7 indole), 6.90–6.97 (m, 3H, 1H of CONH_2_ and indole H-4 and H-6, overlapped with H-4 signal of major diastereomers), 7.27 (br s, 1H, CONH_2_), 10.06 (s, 1H, NH indole), 11.55 (br s, 1H, C(S)NH).

^13^C DEPTQ NMR (101 MHz, DMSO-*d*_6_): the signals of major (*3S,3′S*)/(*3R,3′R*) diastereomers of **16**—20.92* (ArCH_3_), 48.8 (C spiro), 56.59 (C-5′), 59.0* (CH-3′), 109.3* (CH-7 indole), 118.9 (C≡N), 126.6* (CH-4 indole), 127.7 (C-3a indole), 129.4* (CH-6 indole), 130.3 (C-5 indole), 139.7 (C-7a indole), 152.1 (C-6′), 166.6 (CONH_2_), 178.5 (C=O), 198.2 (C=S); the signals of minor (*3S,3′R*)(*3R,3′S*) diastereomers of **16**—20.85* (ArCH_3_), 47.9 (C spiro), 56.64 (C-5′), 60.2* (CH-3′), 109.1* (CH-7 indole), 118.2 (C≡N), 124.1* (CH-4 indole), 129.1* (CH-6 indole), 129.6 (C-3a indole), 129.8 (C-5 indole), 140.5 (C-7a indole), 151.6 (C-6′), 166.9 (CONH_2_), 177.2 (C=O), 199.1 (C=S). *Negatively phased signals.

HRMS (ESI) *m/z*: calculated for C_15_H_14_N_5_O_2_S [M + H]^+^: 328.0868, found 328.0864 (Δ 1.22 ppm).

**Preparation of compounds 17a–d. General procedure**. A 25 mL beaker was charged with 300 mg of thiolates **13a** + **13b** (0.72 mmol) of **14a** + **14b** (0.70 mmol), DMF (5 mL) and water (3 mL). A hot (50 °C) solution of 0.75 mmol of the corresponding alkylating agent in 10 mL EtOH was added to the resulting solution of thiolates **13,14**. A mixture was stirred for another 1 h at 40–50 °C until the reaction completion (TLC) and left for 24 h at room temperature. The precipitated solid was filtered off, washed with EtOH and dried at 60 °C.

*2′-Amino-3′-cyano-5-methyl-2-oxo-6′-{[2-oxo-2-(p-tolylamino)ethyl]thio}-1′H-spiro-[indoline-3,4′-pyridine]-5′-carboxamide* **17a**. Off-white solid, yield 92%.

FTIR, ν_max_, cm^−1^: 3476, 3406, 3358, 3298, 3175 (N–H); 2191 (C≡N); 1678, 1651 (C=O).

^1^H NMR (400 MHz, DMSO-*d*_6_): 2.15 (s, 3H, ArCH_3_), 2.26 (s, 3H, ArCH_3_), 3.86 (*AB-*q, ^2^*J* = 15.3 Hz, 2H, SCH_2_), 5.70 (br s, 2H, NH_2_), 6.54 (d, ^3^*J* = 7.7 Hz, 1H, H-7 indole), 6.75 (br s, 1H, H-4 indole), 6.87 (d, ^3^*J* = 7.7 Hz, 1H, H-6 indole), 7.00 (br s, 1H, CONH_2_), 7.14 (d, ^3^*J* = 8.1 Hz, 2H, H-2 H-6 4-MeC_6_H_4_NH), 7.45–7.49 (m, 3H, 1H CONH_2_ and H-3 H-5 4-MeC_6_H_4_NH overlapped), 8.74 (s, 1H, NH pyridine), 9.90 (s, 1H, NH indole), 10.25 (s, 1H, C(O)NHAr).

^13^C DEPTQ NMR (101 MHz, DMSO-*d*_6_): 20.3* (ArCH_3_), 20.8* (ArCH_3_), 37.3 (SCH_2_), 52.2 (C spiro), 56.4 (C-3′), 108.6* (CH-7 indole), 113.3 (C-5′), 119.7 (C≡N), 119.8* (2C, C-2 C-6 4-MeC_6_H_4_NH), 124.9* (CH-4 indole), 128.4* (CH-6 indole), 129.3* (2C, C-3 C-5 4-MeC_6_H_4_NH), 128.9 (C-Ar), 129.9 (C-Ar), 133.0 (C-Ar), 135.1 (C-Ar), 135.9 (C-Ar), 139.4 (C-2′), 151.9 (C-6′), 166.6 (CONH), 167.1 (CONH), 179.3 (C=O indoline); *Negatively phased signals.

HRMS (ESI) *m/z*: calculated for C_24_H_23_N_6_O_3_S [M + H]^+^: 475.1552, found 475.1551 (Δ 0.21 ppm).

*2′-Amino-3′-cyano-5-methyl-2-oxo-6′-{[2-oxo-2-(o-tolylamino)ethyl]thio}-1′H-spiro-[indoline-3,4′-pyridine]-5′-carboxamide* **17b**. Off-white solid, yield 82%. The compound is sparingly soluble in DMSO. FTIR, ν_max_, cm^−1^: 3437, 3358, 3302, 3283, 3236, 3165 (N–H); 2168 (C≡N); 1699, 1684 (C=O).

^1^H NMR (400 MHz, DMSO-*d*_6_): 2.13 (s, 3H, ArCH_3_), 2.22 (s, 3H, ArCH_3_), 3.94 (*AB-*q, ^2^*J* = 15.4 Hz, 2H, SCH_2_), 5.73 (br s, 2H, NH_2_), 6.54 (d, ^3^*J* = 7.7 Hz, 1H, H-7 indole), 6.77 (br s, 1H, H-4 indole), 6.87 (d, ^3^*J* = 7.7 Hz, 1H, H-6 indole), 6.97 (br s, 1H, CONH_2_), 7.12–7.20 (m, 2H, H-ArNH), 7.25 (d, ^3^*J* = 7.2 Hz, 1H, H-ArNH), 7.36 (d, ^3^*J* = 7.5 Hz, 1H, H-ArNH), 7.52 (br s, 1H, CONH_2_), 8.80 (s, 1H, NH pyridine), 9.80 (s, 1H, NH indole), 9.89 (s, 1H, C(O)NHAr).

^13^C DEPTQ NMR (101 MHz, DMSO-*d*_6_): 17.8* (ArCH_3_), 20.8* (ArCH_3_), 36.6 (SCH_2_), 49.9 (C spiro), 55.9 (C-3′), 108.7* (CH-7 indole), 112.2 (C-5′), 119.7 (C≡N), 124.8* (CH-4 indole), 125.6* (CH ArNH), 126.0* (CH ArNH), 126.1* (CH ArNH), 128.3* (CH-6 indole), 129.8 (C Ar), 129.9 (C Ar), 130.5* (CH ArNH), 133.4 (C-Ar), 135.1 (C-Ar), 135.9 (C-Ar), 138.3 (C-2′), 152.0 (C-6′), 166.6 (CONH), 167.5 (CONH), 179.3 (C=O indoline); *Negatively phased signals.

HRMS (ESI) m/z: calculated for C_24_H_23_N_6_O_3_S [M + H]^+^: 475.1552, found 475.1551 (Δ 0.21 ppm).

*2′-Amino-3′-cyano-5-methyl-2-oxo-6′-[(2-oxo-2-phenylethyl)thio]-1′H-spiro[indoline-3,4′-pyridine]-5′-carboxamide **17c** and 5′-amino-6′-cyano-3′-hydroxy-5-methyl-2-oxo-3′-phenyl- 2′,3′-dihydrospiro[indoline-3,7′-thiazolo[3,2-a]pyridine]-8′-carboxamide* **17c-cycl**. Light brown solid, yield 93%. According to ^1^H NMR, the compound exists as a mixture of linear and cyclic tautomers with a predominance of the **17c-cycl** form in a ratio ~3:7. The compound is sparingly soluble in DMSO. FTIR, ν_max_, cm^−1^: 3441, 3368, 3308, 3234, 3200, 3161 (N–H); 2178 (C≡N); 1697, 1680, 1649 (C=O).

^1^H NMR (400 MHz, DMSO-*d*_6_): the signals of major cyclic tautomeric form **17c-cycl**; signals of **17c-cycl** are doubled due to existence of diastereomeric forms in 1:1 ratio—2.28 (s, 3H, ArCH_3_), 2.31 (s, 3H, ArCH_3_), 3.18–3.22 (two d overlapped, each 1H, SCH_2_ thiazole), 3.54–3.58 (two d overlapped, each 1H, SCH_2_ thiazole), 6.10 (br s, 2H, NH_2_), 6.17 (br s, 2H, NH_2_), 6.72–6.75 (two d overlapped, each 1H, H-7 indole), 6.88–6.89 (m, two s overlapped, each 1H, CONH_2_), 7.03–7.05 (m, 2H CONH_2_ and 2H H-4 indole of both diastereomers **17c-cycl** overlapped), 7.49–7.45 (m, 4H, H-Ar), 7.51–7.52 (m, 4H, H-Ar), 7.56–7.60 (m, 4H, H-Ar), 8.57 (s, 1H, OH), 8.67 (s, 1H, OH), 10.34 (s, 1H, NH indole), 10.35 (s, 1H, NH indole); The observed signals of minor non-cyclic tautomer **17c**—2.23 (s, 3H, ArCH_3_), 4.76 (*AB-*q, ^2^*J* = 17.2 Hz, 2H, SCH_2_C(O)), 5.68 (br s, 2H, NH_2_), 6.56 (d, ^3^*J* = 8.1 Hz, 1H, H-7 indole), 6.99 (br s, 1H, H-4 indole), 7.25 (br s, 1H, CONH_2_), 7.69–7.73 (m, 1H, H-4 C(O)Ph), 8.02 (d, ^3^*J* = 8.3 Hz, 2H, H-2 H-6 C(O)Ph), 8.53 (s, 1H, NH pyridine), 9.91 (s, 1H, NH indole).

^13^C DEPTQ NMR (101 MHz, DMSO-*d*_6_) (all observed signals): 21.22* (CH_3_), 21.24* (CH_3_), 21.3* (CH_3_), 41.4 (SCH_2_), 42.46 (SCH_2_), 43.1 (SCH_2_), 56.4, 110.2*(CH-7 indole), 110.3* (CH-7 indole), 119.5 (CN), 124.0 (C Ar), 125.1* (CH Ar), 125.34* (CH Ar), 125.5* (CH Ar), 128.9* (CH Ar), 129.0* (CH Ar), 129.1* (CH Ar), 129.2* (CH Ar), 129.5* (CH Ar), 129.9* (CH Ar), 130.1 (C Ar), 130.3 (C Ar), 131.8 (C Ar), 131.9 (C Ar), 135.66 (C Ar), 135.68 (C Ar), 142.1 (C Ar), 151.8 (C-6′), 159.9 (C), 167.1 (CONH), 167.8 (CONH), 179.4 (C=O indoline), 179.6 (C=O indoline), 180.5 (C=O indoline), 185.6 (C(O)Ph); *Negatively phased signals.

HRMS (ESI) m/z: calculated for C_23_H_20_N_5_O_3_S [M + H]^+^: 446.1289, found 446.1283 (Δ 1.35 ppm); calculated for C_23_H_19_N_5_NaO_3_S [M + Na]^+^: 468.1106, found 468.1101 (Δ 1.07 ppm).

*2′-Amino-6′-[(2-(4-bromophenyl)-2-oxoethyl)thio]-3′-cyano-2-oxo-1′H-spiro[indoline-3,4′-pyridine]-5′-carboxamide* **17d** *and 5′-amino-3′-(4-bromophenyl)-6′-cyano-3′-hydroxy-2-oxo- 2′,3′-dihydrospiro[indoline-3,7′-thiazolo[3,2-a]pyridine]-8′-carboxamide* **17d-cycl**. Beige solid, yield 92%. According to ^1^H NMR, the compound exists as a mixture of linear and cyclic tautomers with a predominance of the **17d-cycl** form in a ratio ~1:4. The compound is sparingly soluble in DMSO. FTIR, ν_max_, cm^−1^: 3450, 3337, 3312, 3202, 3163 (N–H); 2181 (C≡N); 1701, 1680 (C=O).

^1^H NMR (400 MHz, DMSO-*d*_6_): the signals of major cyclic tautomeric form **17d-cycl**; signals of **17d-cycl** are doubled due to existence of diastereomeric forms in 1:1 ratio—3.19–3.26 (two d overlapped, each 1H, SCH_2_ thiazole), 3.55–3.58 (two d overlapped, each 1H, SCH_2_ thiazole), 6.08 (br s, 2H, NH_2_), 6.14 (br s, 2H, NH_2_), 6.81–6.83 (two d overlapped, each 1H, H-7 indole), 6.99–7.28 (m, 10H, H-Ar, CONH_2_ overlapped), 7.44 (d, ^3^*J* = 8.3 Hz, 2H, H-3 H-5 C(O)Ar), 7.55 (d, ^3^*J* = 8.4 Hz, 2H, H-3 H-5 C(O)Ar), 7.63 (d, ^3^*J* = 8.4 Hz, 2H, H-2 H-6 C(O)Ar), 7.71 (d, ^3^*J* = 8.3 Hz, 2H, H-2 H-6 C(O)Ar), 8.69 (s, 1H, OH), 8.79 (s, 1H, OH), 10.38 (s, 1H, NH indole), 10.41 (s, 1H, NH indole);

The observed signals of minor non-cyclic tautomer **17d**—4.71 (*AB-*q, ^2^*J* = 17.2 Hz, 2H, SCH_2_C(O)), 5.70 (br s, 2H, NH_2_), 6.67 (d, ^3^*J* = 7.6 Hz, 1H, H-7 indole), 6.88–6.92 (m, 1H, H-indole), 7.80 (d, ^3^*J* = 8.3 Hz, 2H, H-3 H-5 C(O)Ar), 7.94 (d, ^3^*J* = 8.3 Hz, 2H, H-2 H-6 C(O)Ar), 8.53 (s, 1H, NH pyridine), 10.02 (s, 1H, NH indole).

^13^C DEPTQ NMR (101 MHz, DMSO-*d*_6_) (all observed signals): 41.8 (SCH_2_), 41.6 (SCH_2_), 42.5 (SCH_2_), 58.8, 61.6, 96.3, 100.0, 108.9* (CH-7 indole), 109.8* (CH-7 indole), 109.9* (CH-7 indole), 118.8 (CN), 118.9 (CN), 121.3* (CH Ar), 121.9 (C Ar), 122.0 (C Ar), 122.1 (C Ar), 122.3* (CH Ar), 122.5* (CH Ar), 124.3* (CH Ar), 124.4* (CH Ar), 127.0* (CH Ar), 127.3* (CH Ar), 128.1* (CH Ar), 128.9* (CH Ar), 129.5 (C Ar), 130.5* (CH Ar), 131.5* (CH Ar), 131.7* (CH Ar), 132.1* (CH Ar), 134.2 (C Ar), 134.5 (C Ar), 134.7 (C Ar), 134.8 (C Ar), 1401.0 (C Ar), 141.1 (C Ar), 141.6 (C Ar), 141.9 (C Ar), 146.6 (C Ar), 151.2 (C), 151.6 (C), 152.0 (C), 167.1 (CONH), 167.3 (CONH), 178.49 (C=O indoline), 179.1 (C=O indoline), 188.7 (C(O)Ar); *Negatively phased signals.

HRMS (ESI) m/z: calculated for C_22_H_17_BrN_5_O_3_S [M + H]^+^: 510.0236, found 510.0235 (Δ 0.1 ppm); calculated for C_22_H_15_BrN_5_O_2_S [M + H–H_2_O]^+^: 492.0130, found 492.0130 (Δ 0 ppm).

### 3.1. Antibacterial Studies

A suspension of pathogens isolated from a swollen ear canal was prepared by transferring a loop full of isolated colony to 5 mL of normal saline in tubes. The turbidity of the bacterial growth in tubes was then compared with the turbidity of 0.5 McFarland suspension to give an approximate number of live cells equal to 0.5 × 10^8^ CFU/mL. Then, 0.1 mL of the bacterial suspension was transferred and spread homogeneously over the Mueller–Hinton agar with a sterile swab and the plate was allowed to stand for 10 min at room temperature to dry. Using a sterile cork borer, a 6 mm diameter hole was made in each prepared plate. A solution of compound **17a** at 100 mg/mL concentration was prepared by dissolving **17a** in dimethyl sulfoxide (DMSO). Then, 100 μL of the solution was added using a micropipette in each hole under sterile conditions; DMSO was also added as control. The plate was kept for 24 h at 37 °C in an incubator. The diameter of the inhibitory zone around each hole was measured in millimeters.

The minimum inhibitory concentration (MIC) against *P. aeruginosa* ATCC 27,853 was determined using the classical broth dilution method [101] by preparing differently concentrated solutions of **17a** using nutrient broth along with positive and negative controls for each pathogenic bacteria. The turbidity of *P. aeruginosa* suspension was brought to 0.5 McFarland and 100 μL of bacterial suspension was added to the solutions of **17a**. The positive control consisted of 1 mL of nutrient broth and 100 μL of bacterial suspension, and 1 mL of nutrient broth was used only as a negative control. All tubes were incubated at 37 °C for 24 h. After incubation, 100 μL of suspension was dropped onto sterile Mueller–Hinton agar and the plates were incubated at 37 °C for 24 h.

### 3.2. Antioxidant Activity of Compound ***17a***

The solutions of different concentrations (100,000, 10,000, 1000 and 100 µg/mL) were prepared from compound **17a**. Methanolic solution of DPPH was prepared by dissolving 4 mg of DPPH in 100mL of MeOH. Then, 1 mL of DPPH methanolic solution was added to 1 mL of each solution of **17a**. The reaction mixture was incubated for 30 min in the dark at room temperature. The absorption of the resulting solutions was recorded at 517 nm. The DPPH color changes from deep violet (free radical form) to pale yellow (1,1-diphenyl-2-picryl hydrazine, reduced form). Ascorbic acid was used as a positive control and methanolic solution was used as a negative control.

### 3.3. Herbicide-Safening Effect Studies

The germinated sunflower seeds (cv. Master) with 2–4 mm long embryo roots were placed in a solution of 2,4-D (10^−3^ % by weight) for 1 h to achieve 40–60% inhibition of hypocotyl growth. After treatment, the seedlings were washed with pure water and placed into a solution of the corresponding compound **16a** and **17a,b** (concentrations 10^−2^, 10^−3^, 10^−4^ or 10^−5^% by weight, “herbicide + antidote” experiments). After 1 h, the seedlings were washed with pure water and placed on paper strips (10 × 75 cm, 20 seeds per strip). The strips were rolled and placed into beakers with water (50 cm^3^). The reference group of seedlings (“herbicide” experiments) was kept in 2,4-D solution (10^−3^%) for 1 h and then in water for 1 h. The “control” seedlings were kept in water for 2 h. The temperature of all solutions was maintained at 28 °C. The seedlings were then thermostated for 3 days at 28 °C. Each experiment was performed in triplicate; 20 seeds were used in each experiment. The results are given in Table 3.

## 4. Conclusions

In summary, we studied for the first time the reaction between isatin, malononitrile and monothiomalonamide in the presence of triethylamine. It was found that the reaction proceeds with the formation of two isomeric 1′H and 3′H products—triethylammonium 6′-amino-3′-(aminocarbonyl)-5′-cyano-2-oxo-1,2-dihydro-1′H-spiro-[indole-3,4′-pyridine]-2′-thiolate and 6′-amino-3′-(aminocarbonyl)-5′-cyano-2-oxo-1,2-dihydro-3′H-spiro-[indole-3,4′-pyridine]-2′-thiolate. These results are in good agreement with the results of other works on the preparation of related nicotinamides, starting from monothiomalonamide. We failed to oxidize the above thiolates to isothiazolo[5,4-b]pyridines upon treatment with DMSO-HCl system. Instead, diastereomers of 6′-amino-5′-cyano-5-methyl-2-oxo-2′-thioxo-1,2,2′,3′-tetrahydro-1′H- spiro[indole-3,4′-pyridine]-3′-carboxamide were isolated. The alkylation of a mixture of the above 1′H/3′H thiolates proceeded in a regionspecific way on the sulfur atom and led to the formation of only 1′H derivatives in high yields. In the case of the reaction involving α-bromoketones, the isolated alkylation products were found to exist as a mixture of linear and chained (thiazolo[3,2-a]pyridine) tautomers. The structure of the new compounds was thoroughly investigated using IR, HRMS and NMR spectroscopy, including DEPTQ and 2D ^1^H-^13^C HSQC and ^1^H-^13^C HMBC experiments. Molecular docking studies for some of the new compounds against the regulatory protein PqsR of *Pseudomonas aeruginosa* showed promising results. Among the most active compounds, both enantiomers of 2′-amino-3′-cyano-5-methyl-2-oxo-6′-{[2-oxo-2-(p-tolylamino)ethyl]thio}- 1′H-spiro[indoline-3,4′-pyridine]-5′-carboxamide **17a** were recognized. The antibacterial effect of **17a** was confirmed in experiments *in vitro*. Additionally, compound **17a** revealed a pronounced antioxidant effect comparable with that for ascorbic acid in the experiments with 1,1-diphenyl-2-picrylhydrazyl radical. Finally, spiro[indole-3,4′-pyridines] **16a** and **17a,b** were recognized as moderately active 2,4-D herbicide safeners and reduced the negative effect of 2,4-D on sunflower seedling hypocotyls by 16–33% and by 12–25% on sunflower seedling roots.

## Figures and Tables

**Table 1:** The results obtained from the rigid docking studies for stereoisomers of compounds **16,17a,b** against bacterial transcriptional regulator protein PqsR of *Pseudomonas aeruginosa* (PDB ID 4JVC).

N	Compound	Docking Score, kcal/mol
1	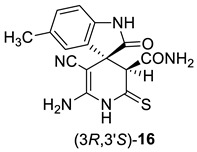	−6.2
2	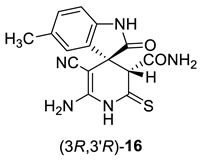	−6.7
3	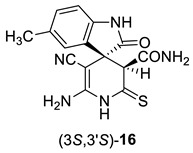	−5.8
4	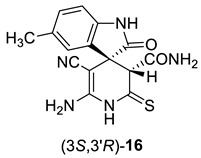	−6.1
5	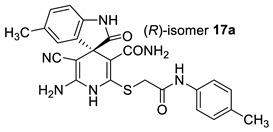	−8.2
6	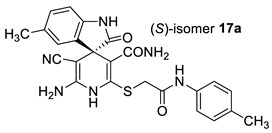	−8.0
7	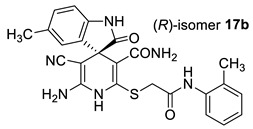	−7.3
8	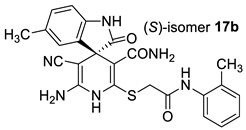	−6.7

**Table 2:** Percentage of free radical inhibition of **17a** against DPPH compared with the control (ascorbic acid).

	Sample	Concentration of 17a, µg/mL			
		100,000	10,000	1000	100
Inhibition, *I* (%)	Ascorbic acid	66	74	78	86
	**17a**	28	83	90	88

**Table 3:** The antidote effects of the most active compounds **16a** and **17a,b** with respect to herbicide 2,4-D.

N	Compound	Organ	Antidote Effect *A* at Different Concentrations, % ^1^			
			10^−2^	10^−3^	10^−4^	10^−5^
1	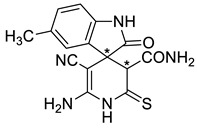 **16a**	roots	123	121	125	122
		hypocotyls	127	130	124	127
2	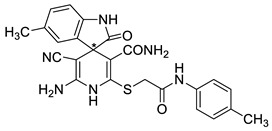 **17a**	roots	114	120	117	117
		hypocotyls	130	129	133	131
3	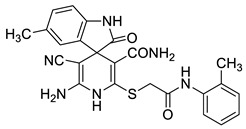 **17b**	roots	112	119	125	114
		hypocotyls	117	116	119	120

^1^ The differences are reliable at *p* = 0.95.

## Data Availability

File Electronic Supplementary Material.pdf containing ^1^H and ^13^C DEPTQ NMR, 2D NMR ^1^H-^13^C HSQC and ^1^H-^13^C HMBC, FTIR, HRMS spectral charts for new compounds (Figures S1–S39, Tables S1 and S2).

## Supplementary Materials

The following supporting information can be downloaded at: https://www.mdpi.com/article/10.3390/molecules28073161/s1. ^1^H and ^13^C DEPTQ NMR, 2D NMR ^1^H-^13^C HSQC and ^1^H-^13^C HMBC, FTIR, HRMS spectral charts for new compounds (Figures S1–S39, Tables S1 and S2).

## Sample Availability

Samples of the compounds are available from the authors.
