# Supplementary material for: New 6′-Amino-5′-cyano-2-oxo-1,2-dihydro-1′H-spiro[indole-3,4′-pyridine]-3′-carboxamides: Synthesis, Reactions, Molecular Docking Studies and Biological Activity
Source: Molecules. 2023 Apr 2;28(7):3161. doi: 10.3390/molecules28073161 (PMC10096136; doi:10.3390/molecules28073161)

Electronic Supplementary Material file

New 6'-amino-5'-cyano-2-oxo-1,2-dihydro-1'*H*-spiro[indole-3,4'-pyridine]-3'-carboxamides: synthesis, reactions, molecular docking studies and biological activity

Victor V. Dotsenko[1,2,*], Nawras T. Jassim[1], Azamat Z. Temerdashev[3], Zainab R. Abdul-Hussein[4], Nicolai A. Aksenov[2] and Inna V. Aksenova [2]

[1]Department of Organic Chemistry and Technologies, Kuban State University, 149 Stavropolskaya St., 350040 Krasnodar, Russia;
[2]Department of Chemistry, North Caucasus Federal University, 1a Pushkin St., 355017 Stavropol, Russia;
[3]Department of Analytical Chemistry, Kuban State University, 149 Stavropolskaya St., 350040 Krasnodar, Russia;
[4]Department of Pathological Analysis, College of Science, University of Basrah, PO Box 49, 61004 Basrah, Iraq

*Correspondence: victor_dotsenko_@mail.ru (V.V.D.).

Contents

Figure S1. FTIR spectrum of a mixture of 13a and 13b 3
Figure S2. ^{1}H NMR spectrum of a mixture of 13a and 13b, DMSO-d_6 (400 MHz) 3
Figure S3. ^{13}C DEPTQ NMR spectrum of a mixture of 13a and 13b, DMSO-d_6 (101 MHz) ... 4
Figure S4. ^{1}H–^{13}C HSQC NMR spectrum of a mixture of 13a and 13b, DMSO-d_6 (400/101 MHz) 4
Figure S5. ^{1}H–^{13}C HSQC NMR spectrum of a mixture of 13a and 13b, DMSO-d_6 (400/101 MHz) (fragment) 5
Figure S6. ^{1}H–^{13}C HMBC NMR spectrum of a mixture of 13a and 13b, DMSO-d_6 (400/101 MHz) 5
Table S1. The observed correlations in the ^{1}H-^{13}C HSQC and ^{1}H-^{13}C HMBC 2D NMR spectra of thiolate 13a 6
Figure S7. FTIR spectrum of a mixture of 14a and 14b 7
Figure S8. ^{1}H NMR spectrum of a mixture of 14a and 14b, DMSO-d_6 (400 MHz) 7
Figure S9. ^{13}C DEPTQ NMR spectrum of a mixture of 14a and 14b, DMSO-d_6 (101 MHz) ... 8
Figure S10. ^{1}H–^{13}C HSQC NMR spectrum of a mixture of 14a and 14b, DMSO-d_6 (400/101 MHz) 8
Figure S11. ^{1}H–^{13}C HSQC NMR spectrum of a mixture of 14a and 14b, DMSO-d6 (400/101 MHz) (*fragment*) 9
Figure S12. ^{1}H–^{13}C HSQC NMR spectrum of a mixture of 14a and 14b, DMSO-d6 (400/101 MHz) (*fragment*) 9
Figure S13. ^{1}H–^{13}C HMBC NMR spectrum of a mixture of 14a and 14b, DMSO-d_6 (400/101 MHz) 10

Figure S14. ^{1}H–^{13}C HMBC NMR spectrum of a mixture of 14a and 14b, DMSO-d_6 (400/101 MHz) (*fragments*) 11
Figure S15. ^{1}H–^{13}C HMBC NMR spectrum of a mixture of 14a and 14b, DMSO-d_6 (400/101 MHz) (*fragments*) 12
Table S2. The observed correlations in the ^{1}H-^{13}C HSQC and ^{1}H-^{13}C HMBC 2D NMR spectra of thiolate 14a (major 1'H-isomer) 13
Figure S16. FTIR spectrum of 6'-amino-5'-cyano-5-methyl-2-oxo-2'-thioxo-1,2,2',3'-tetrahydro-1'H-spiro[indole-3,4'-pyridine]-3'-carboxamide 16 14
Figure S17. ^{1}H NMR spectrum of 6'-amino-5'-cyano-5-methyl-2-oxo-2'-thioxo-1,2,2',3'-tetrahydro-1'H-spiro[indole-3,4'-pyridine]-3'-carboxamide 16, DMSO-d_6 (400 MHz) 14
Figure S18. ^{13}C DEPTQ NMR spectrum of 6'-amino-5'-cyano-5-methyl-2-oxo-2'-thioxo-1,2,2',3'-tetrahydro-1'H-spiro[indole-3,4'-pyridine]-3'-carboxamide 16, DMSO-d_6 (101 MHz)15
Figure S19. FTIR spectrum of compound 17a 15
Figure S20. ^{1}H NMR spectrum of compound 17a, DMSO-d_6 (400 MHz) 16
Figure S21. ^{13}C DEPTQ NMR spectrum of compound 17a, DMSO-d_6 (101 MHz) 16
Figure S22. FTIR spectrum of compound 17b 17
Figure S23. ^{1}H NMR spectrum of compound 17b, DMSO-d_6 (400 MHz) 17
Figure S24. ^{13}C DEPTQ NMR spectrum of compound 17b, DMSO-d_6 (101 MHz) 18
Figure S25. FTIR spectrum of compound 17c+17c-cycl 18
Figure S26. ^{1}H NMR spectrum of compound 17c+17c-cycl, DMSO-d_6 (400 MHz) 19
Figure S27. ^{1}H NMR spectrum of compound 17c+17c-cycl, DMSO-d_6 (400 MHz) (*fragment*)19
Figure S28. ^{13}C DEPTQ NMR spectrum of compound 17c+17c-cycl, DMSO-d_6 (101 MHz) 20
Figure S29. FTIR spectrum of compound 17d+17d-cycl 20
Figure S30. ^{1}H NMR spectrum of compound 17d+17d-cycl, DMSO-d_6 (400 MHz) 21
Figure S31. ^{1}H NMR spectrum of compound 17d+17d-cycl, DMSO-d_6 (400 MHz) (*fragment*)21
Figure S32. ^{13}C DEPTQ NMR spectrum of compound 17d+17d-cycl, DMSO-d_6 (101 MHz)22
Figure S33. HRMS (ESI) spectrum of thiolates 13a+13b 23
Figure S34. HRMS spectrum of thiolates 14a+14b 23
Figure S35. HRMS spectrum of 6'-amino-5'-cyano-5-methyl-2-oxo-2'-thioxo-1,2,2',3'-tetrahydro-1'H- spiro[indole-3,4'-pyridine]-3'-carboxamide 16 24
Figure S36. HRMS spectrum of compound 17a 24
Figure S37. HRMS spectrum of compound 17b 25
Figure S38. HRMS spectrum of compound 17c+17c-cycl 25
Figure S39. HRMS spectrum of compound 17d+17d-cycl 26

Figure S1. FTIR spectrum of a mixture of 13a and 13b

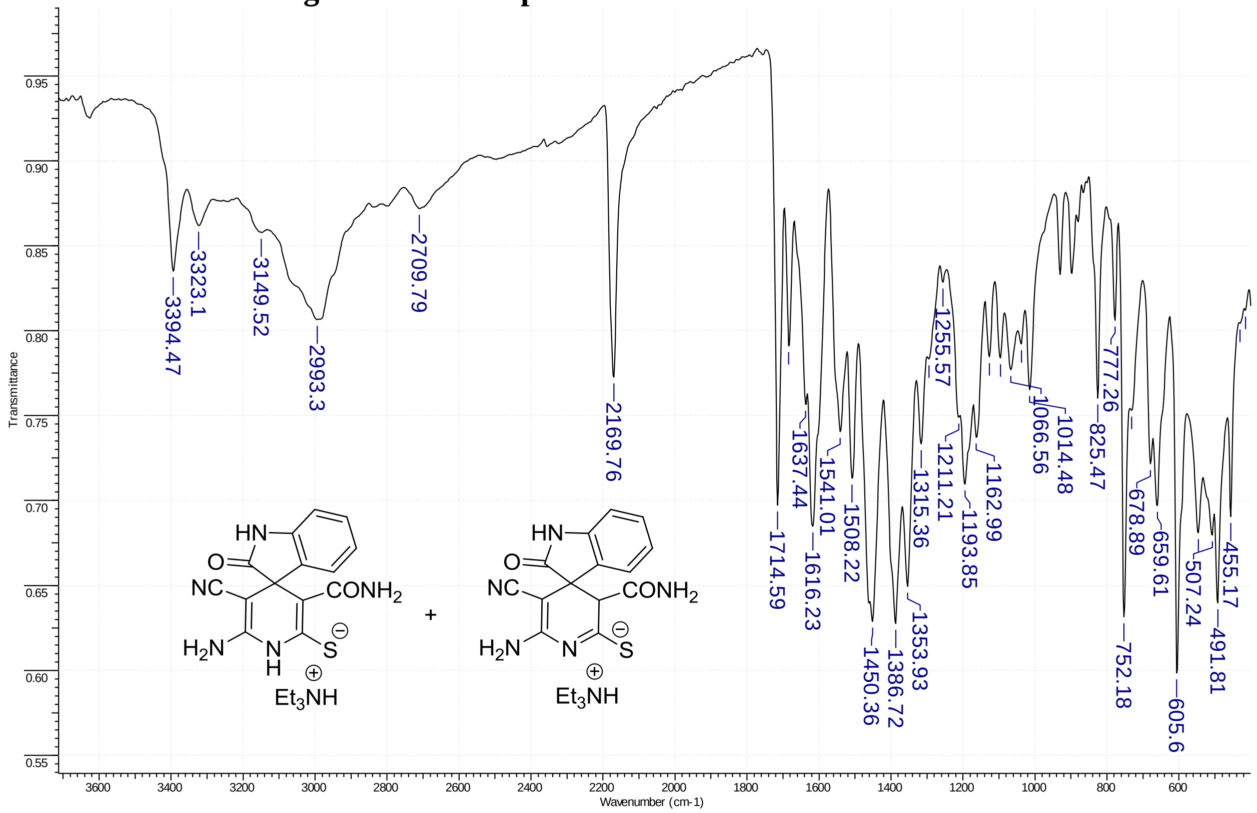

Figure S2. ^{1}H NMR spectrum of a mixture of 13a and 13b, DMSO-d_6 (400 MHz)

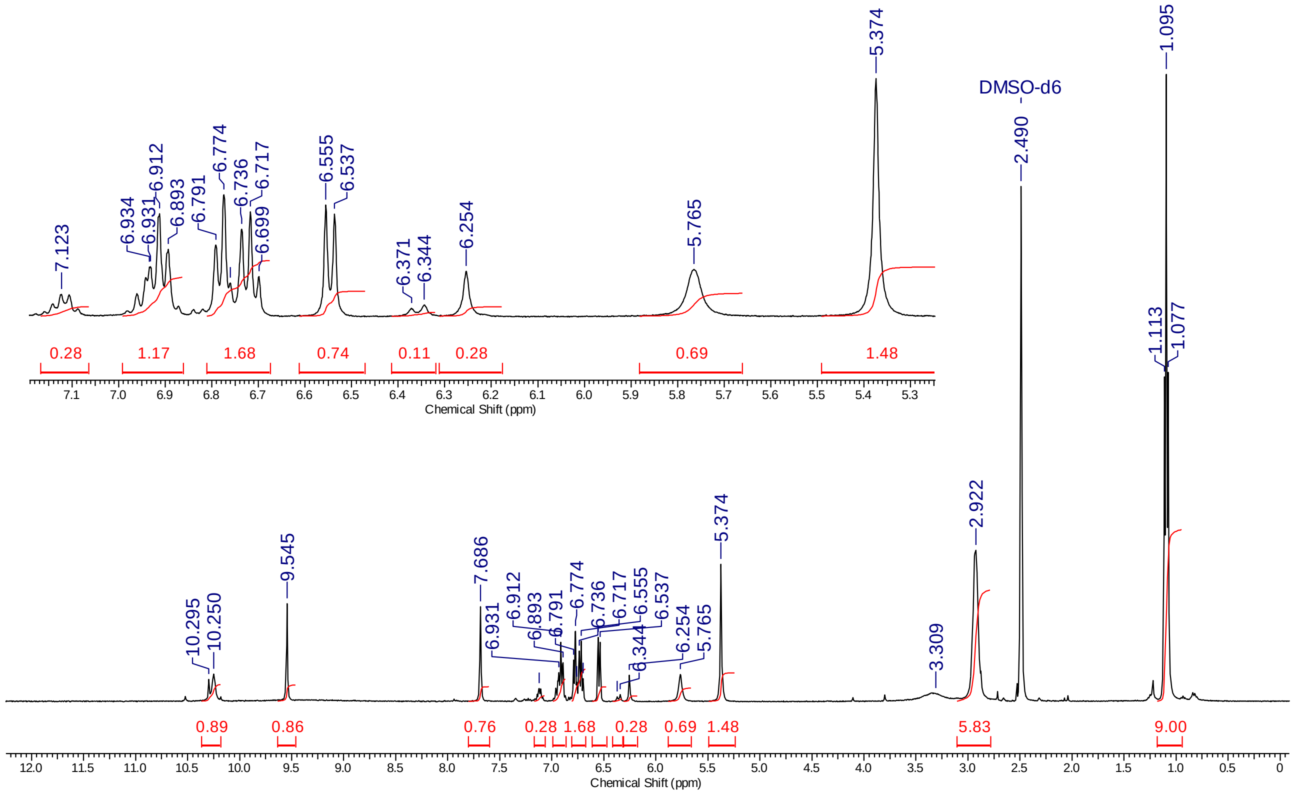

Figure S3. ^{13}C **DEPTQ NMR spectrum of a mixture of 13a and 13b, DMSO-d$_6$ (101 MHz)**

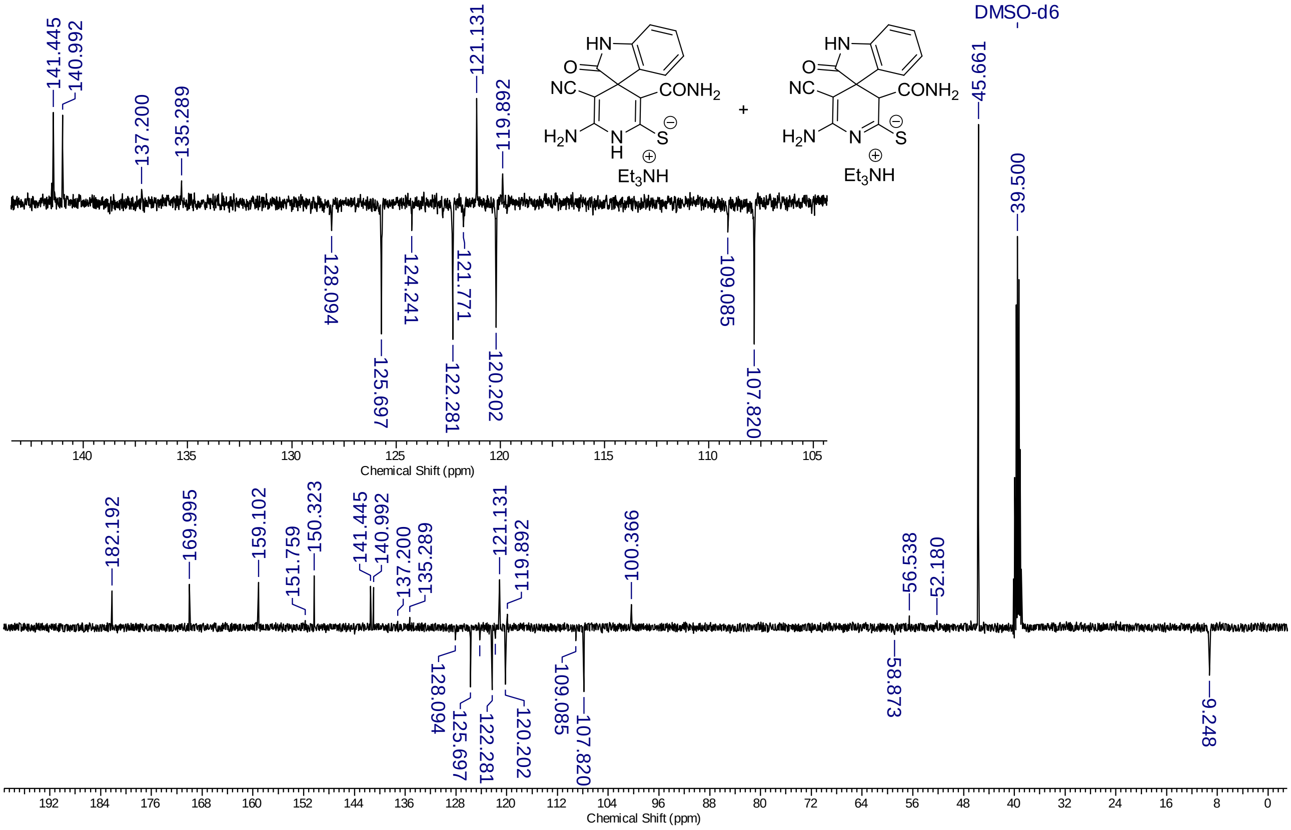

Figure S4. ^{1}H–^{13}C **HSQC NMR spectrum of a mixture of 13a and 13b, DMSO-d$_6$ (400/101 MHz)**

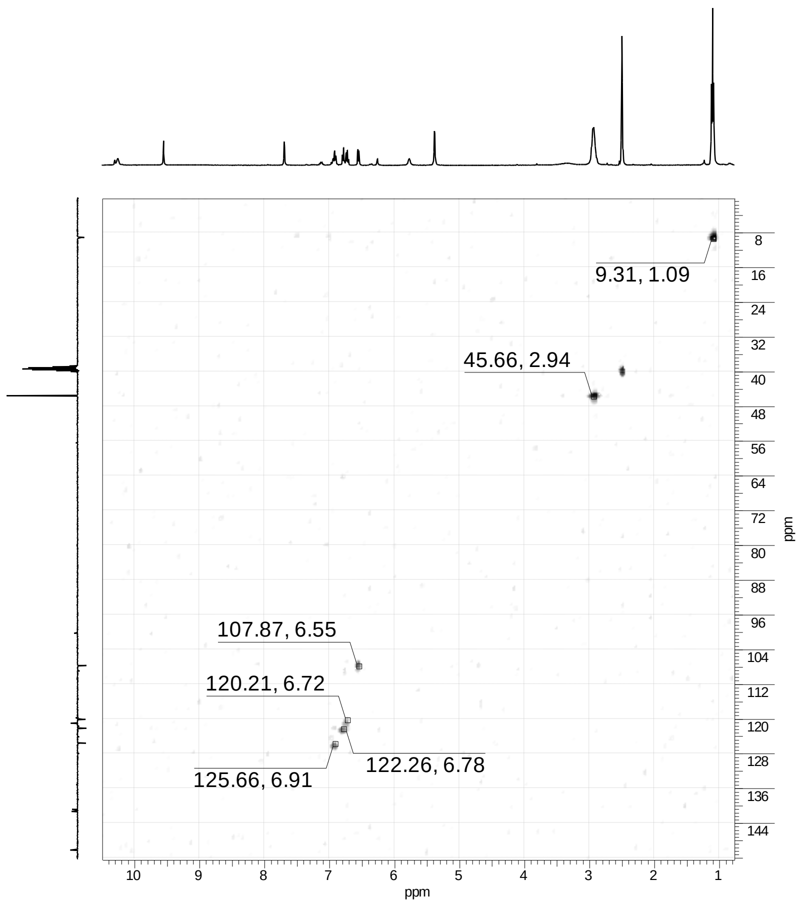

Figure S5. ^{1}H–^{13}C HSQC NMR spectrum of a mixture of 13a and 13b, DMSO-d_6 (400/101 MHz) (fragment)

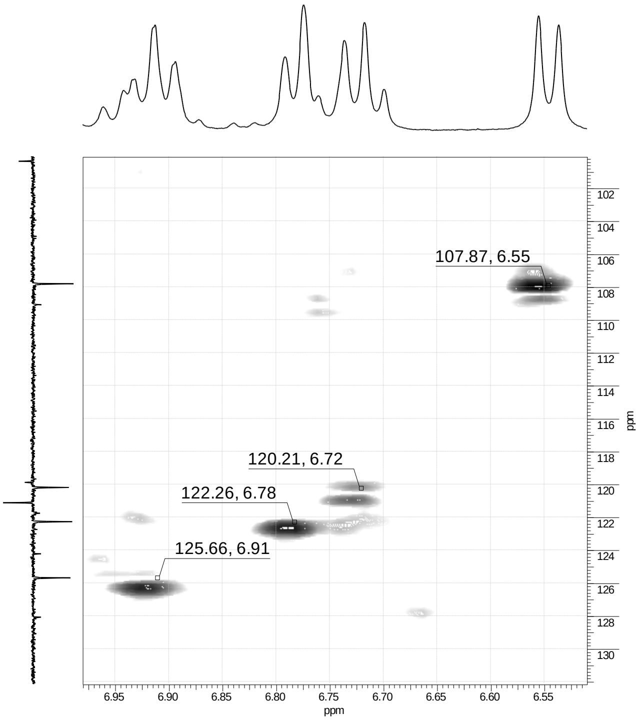

Figure S6. ^{1}H–^{13}C HMBC NMR spectrum of a mixture of 13a and 13b, DMSO-d_6 (400/101 MHz)

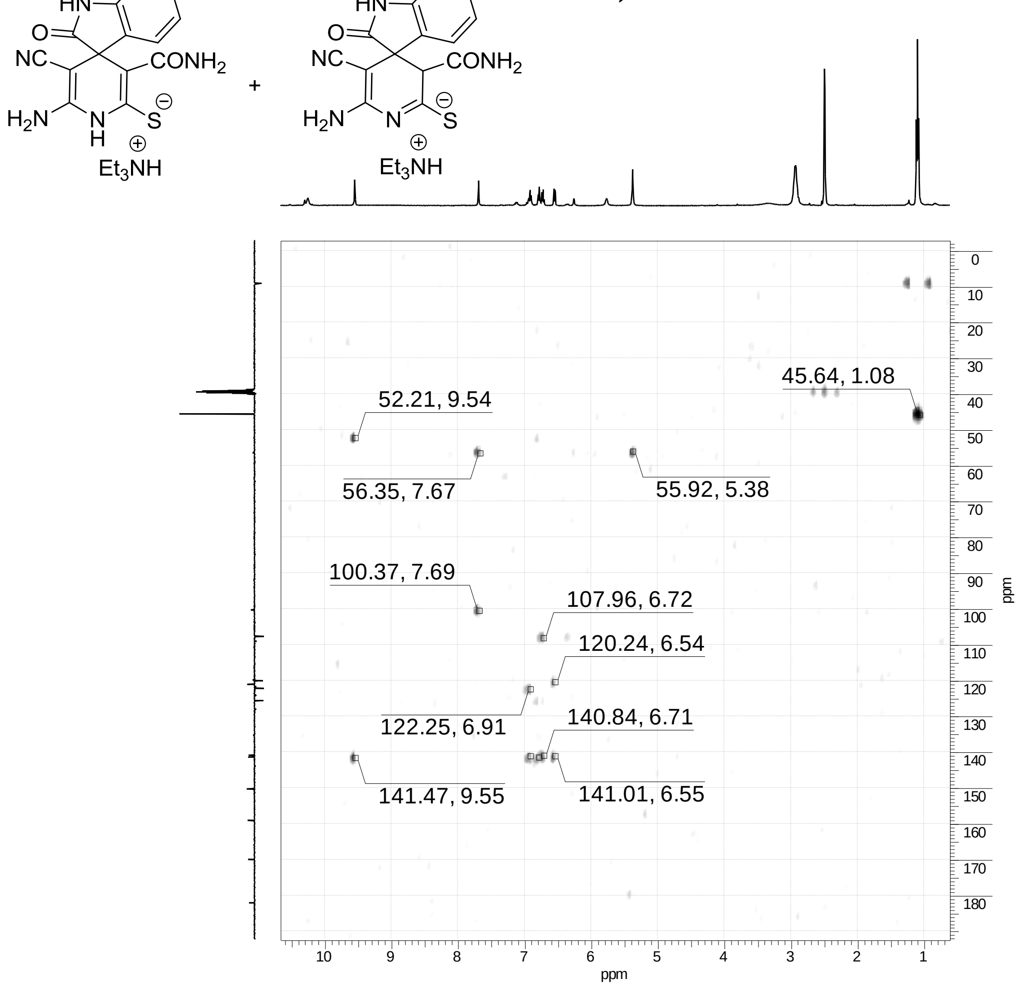

Table S1. The observed correlations in the ^{1}H-^{13}C HSQC and ^{1}H-^{13}C HMBC 2D NMR spectra of thiolate 13a

^{13}C chemical shifts are given in **red**, ^{1}H shifts – in **blue**

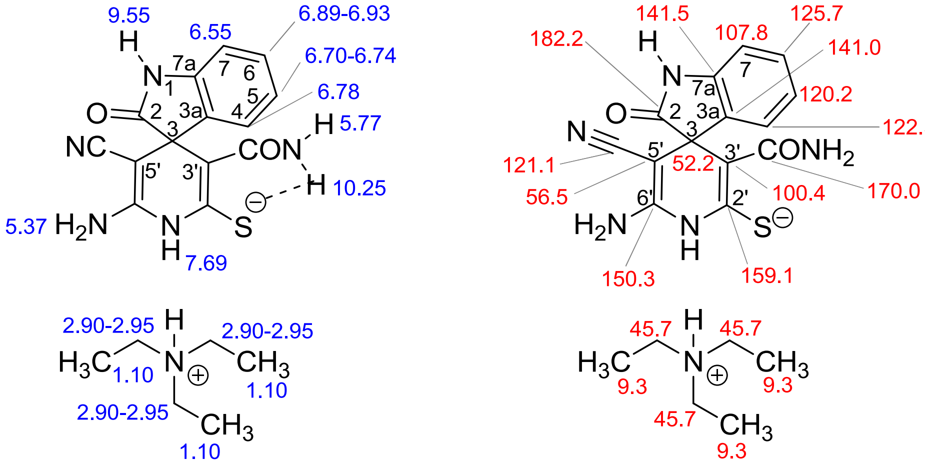

^{1}H NMR shifts, δ, ppm	Correlations in HSQC spectrum, δ, ppm	Correlations in HMBC spectrum, δ, ppm
1.10 (t, ^{3}J = 7.2 Hz, 9H, 3 $C\underline{H}_3CH_2N$)	9.3* ($NCH_2\underline{C}H_3$)	45.7 ($N\underline{C}H_2CH_3$)
2.90–2.95 (m, 6H, 3 $C\underline{H}_3CH_2N$)	45.7 ($N\underline{C}H_2CH_3$)	–
5.37 (br s, 2H, NH_2)	–	56.5 (C-5')
5.77 (br s, 1H, $C(O)NH_2$)	–	–
6.55 (d, ^{3}J = 7.5 Hz, 1H, H-7 indole)	107.8* (CH-7 indole)	120.2* (CH-5 indole), 141.0 (C-3a indole)
6.70–6.74 (m, 1H, H-5 indole)	120.2* (CH-5 indole)	107.8* (CH-7 indole), 141.0 (C-3a indole)
6.78 (d, ^{3}J = 7.0 Hz, 1H, H-4 indole)	122.3* (CH-4 indole)	125.7* (CH-6 indole), 141.5 (C-7a indole)
6.89–6.93 (m, 1H, H-6 indole)	125.7* (CH-6 indole)	122.3* (CH-4 indole), 141.5 (C-7a indole)
7.69 (s, 1H, NH pyridine)	–	56.5 (C-5'), 100.4 (C-3')
9.55 (s, 1H, NH indole)	–	52.2 (C spiro), 141.0 (C-3a indole)
10.25 (br s, 1H, $C(O)NH_2$)	–	–

*Signals with a negative phase.

Figure S7. FTIR spectrum of a mixture of 14a and 14b

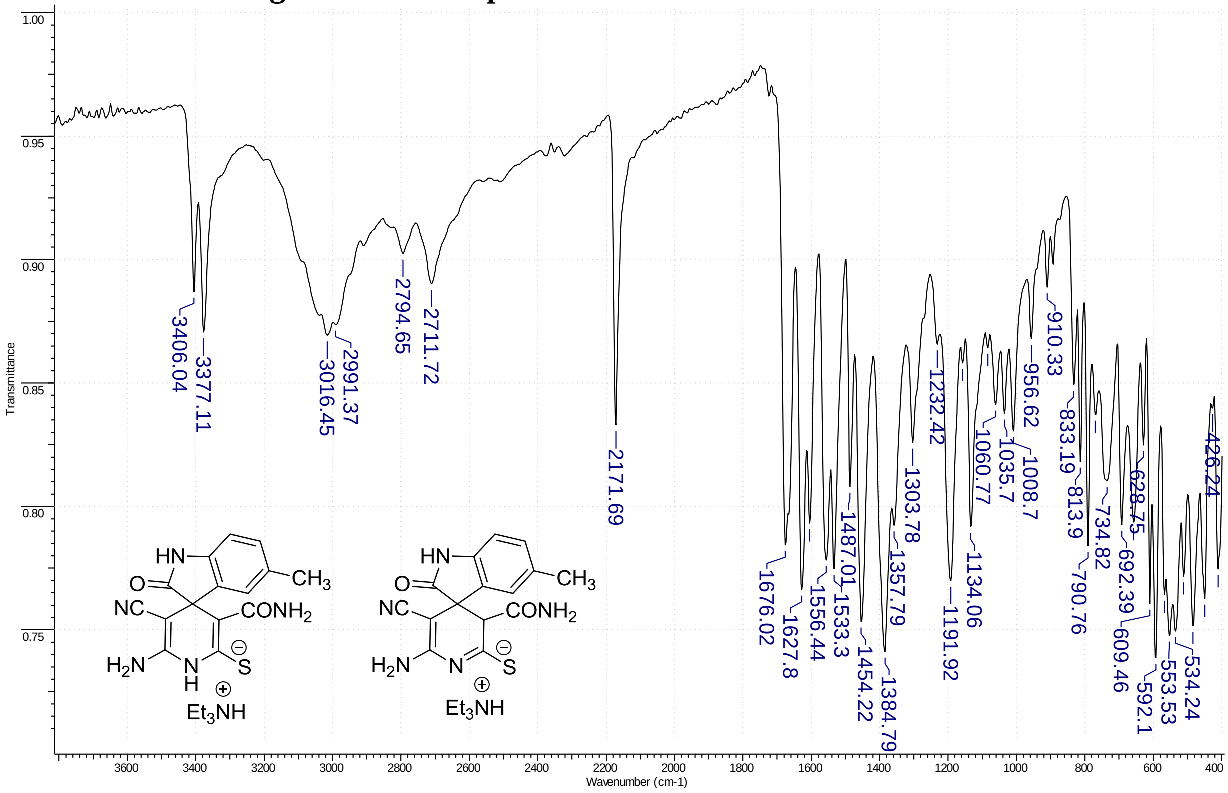

Figure S8. ^{1}H NMR spectrum of a mixture of 14a and 14b, DMSO-d_6 (400 MHz)

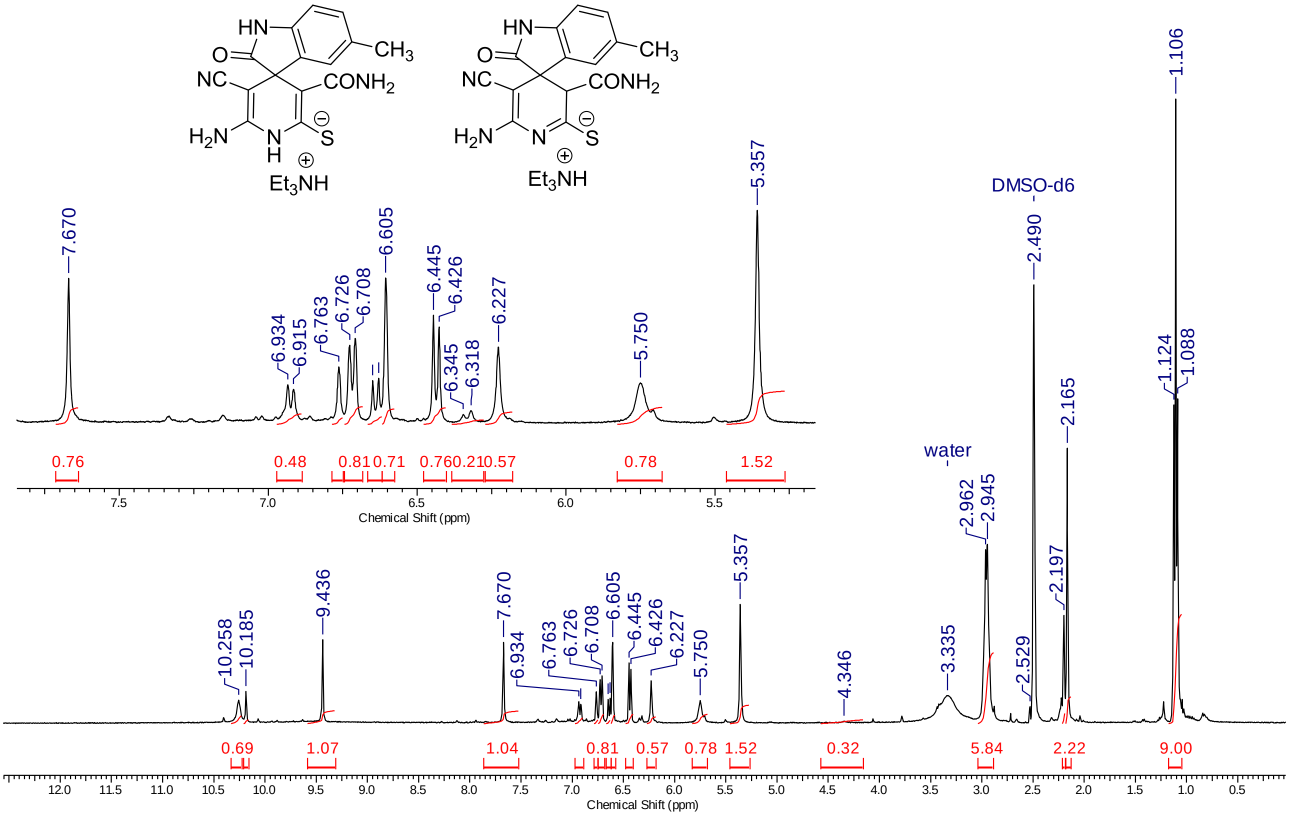

Figure S9. ^{13}**C DEPTQ NMR spectrum of a mixture of 14a and 14b, DMSO-d$_6$ (101 MHz)**

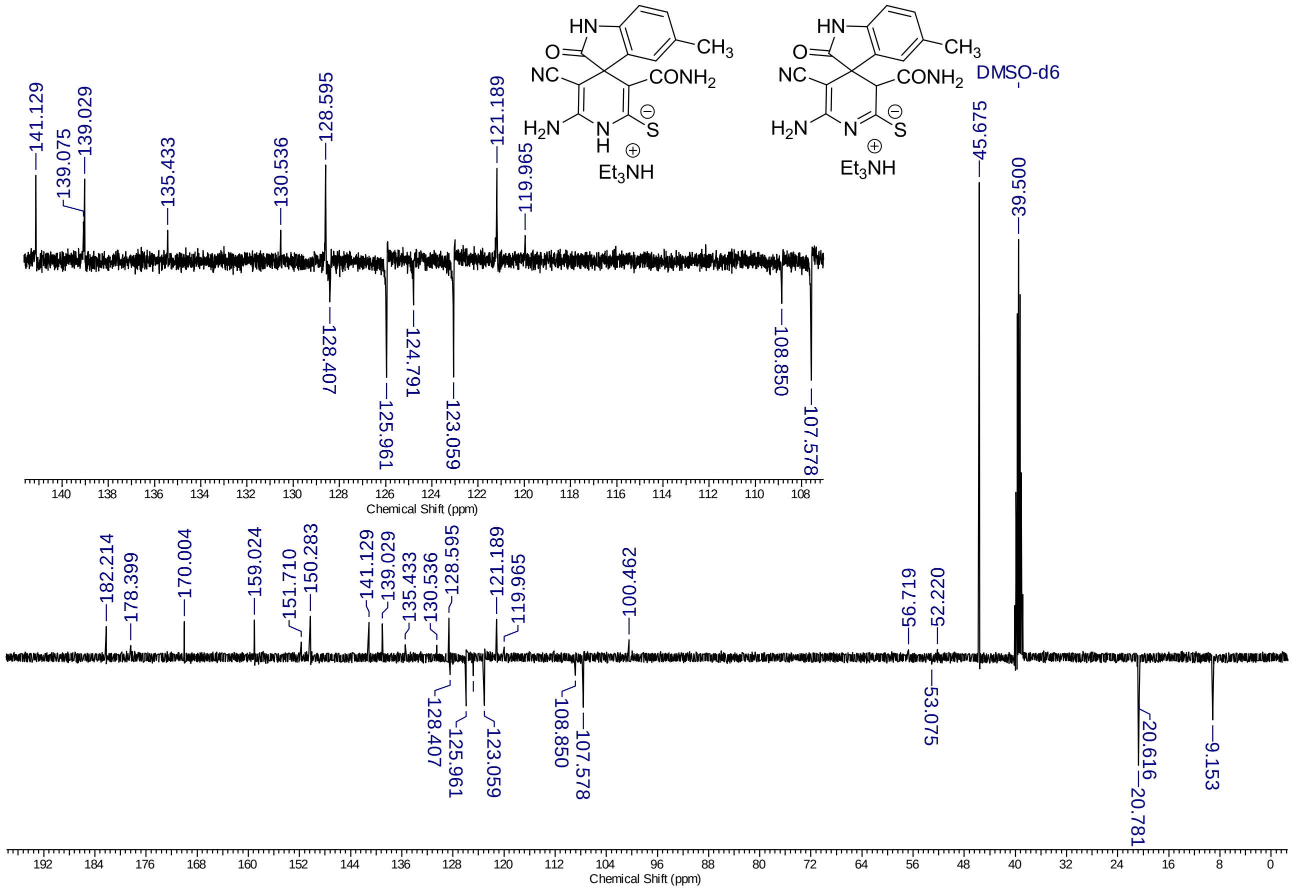

Figure S10. ^{1}**H–**^{13}**C HSQC NMR spectrum of a mixture of 14a and 14b, DMSO-d$_6$ (400/101 MHz)**

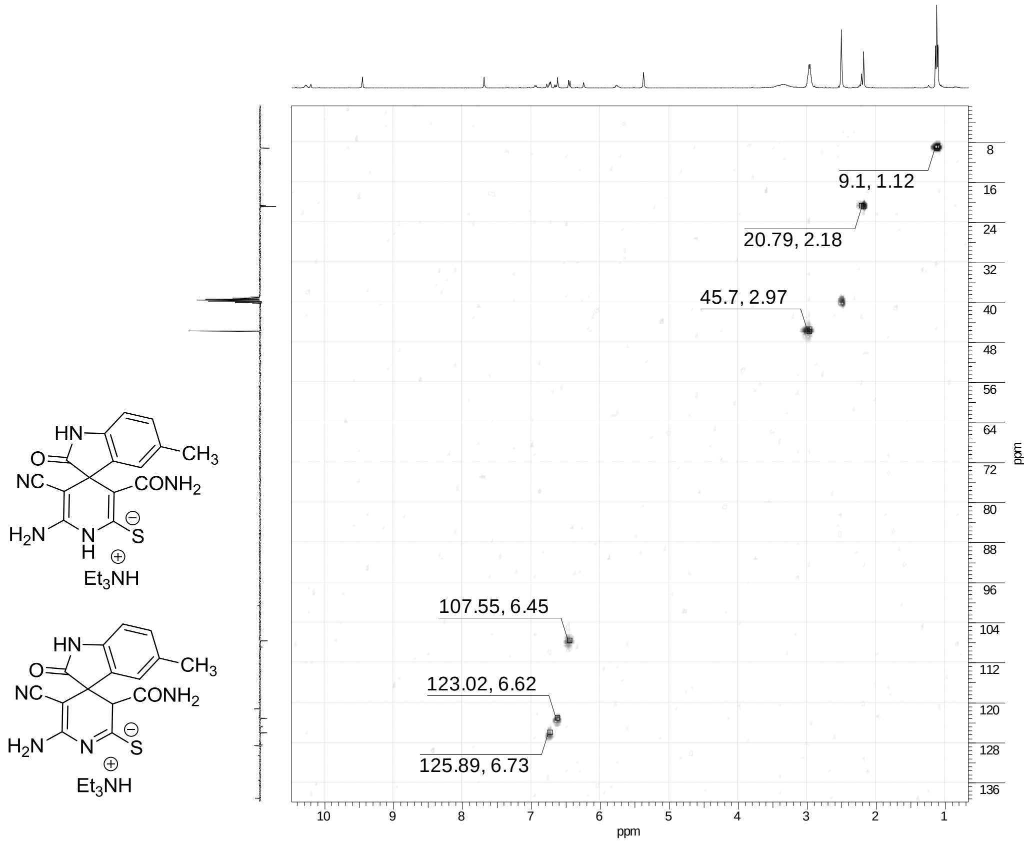

Figure S11. ^{1}H–^{13}C HSQC NMR spectrum of a mixture of 14a and 14b, DMSO-d6 (400/101 MHz) (*fragment*)

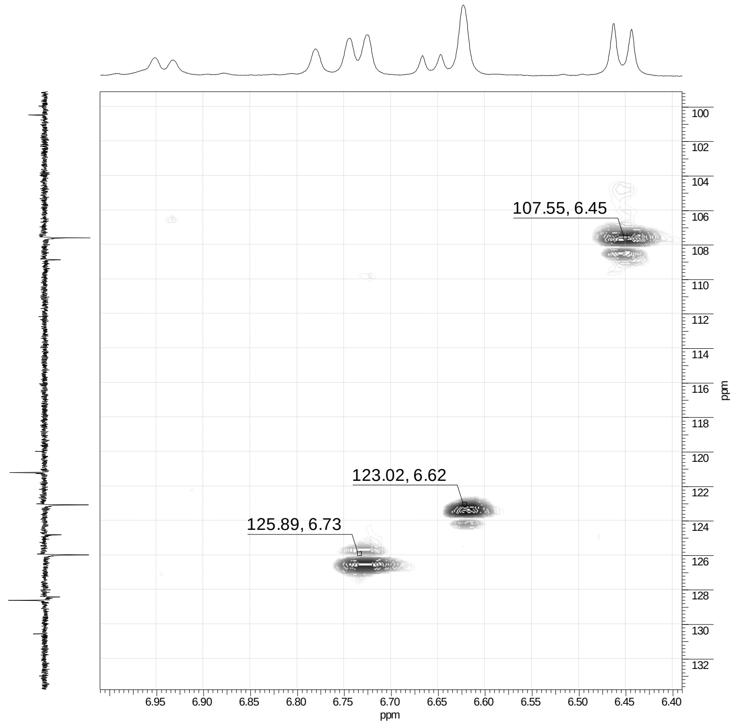

Figure S12. ^{1}H–^{13}C HSQC NMR spectrum of a mixture of 14a and 14b, DMSO-d6 (400/101 MHz) (*fragment*)

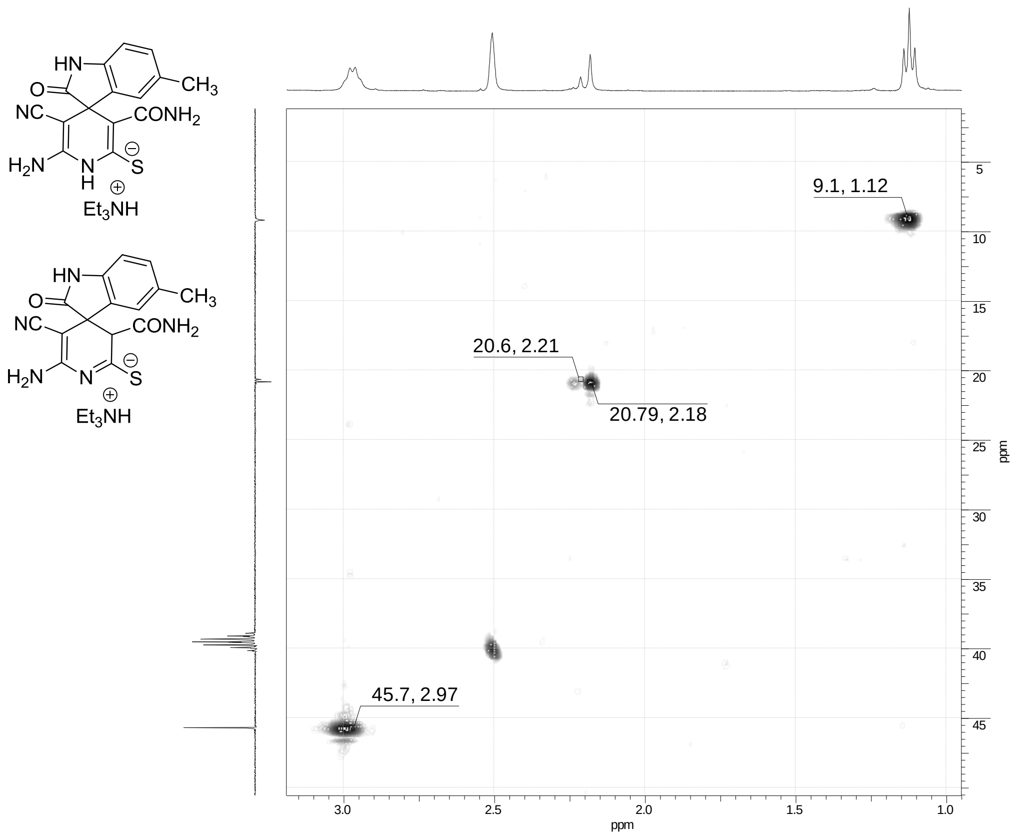

Figure S13. ^{1}H–^{13}C HMBC NMR spectrum of a mixture of 14a and 14b, DMSO-d_6 (400/101 MHz)

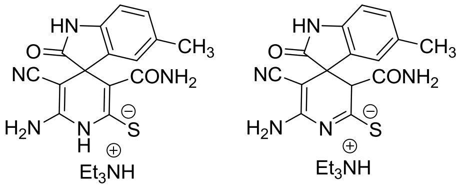

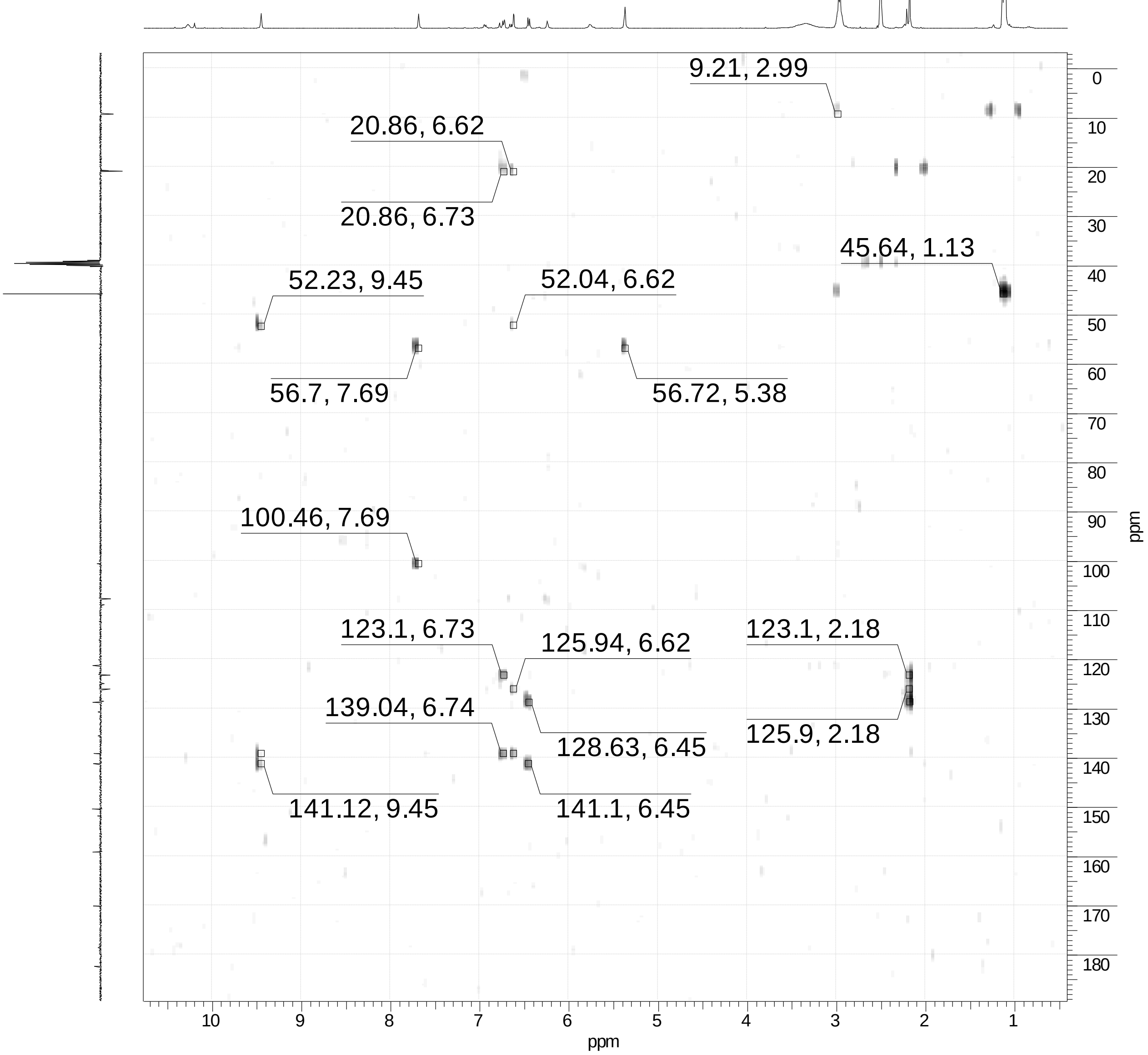

Figure S14. ^{1}H–^{13}C HMBC NMR spectrum of a mixture of 14a and 14b, DMSO-d_6 (400/101 MHz) (*fragments*)

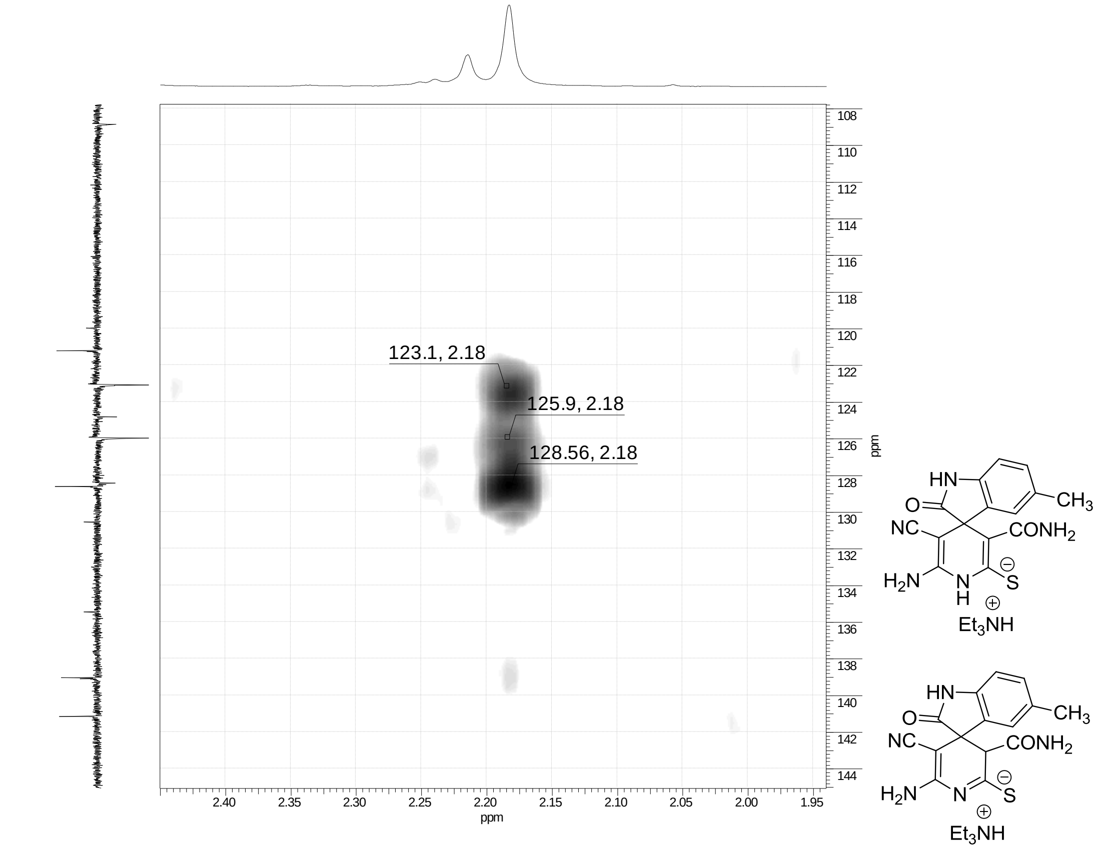

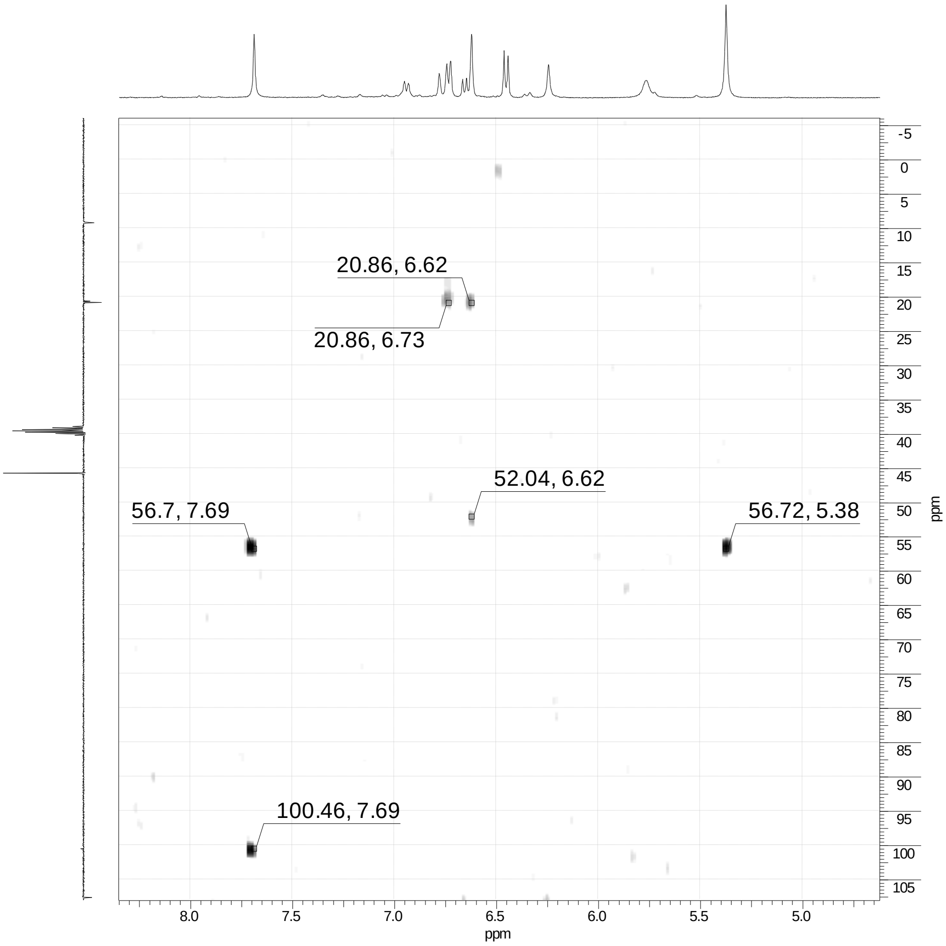

Figure S15. ^{1}H–^{13}C **HMBC NMR spectrum of a mixture of 14a and 14b, DMSO-d_6 (400/101 MHz) (*fragments*)**

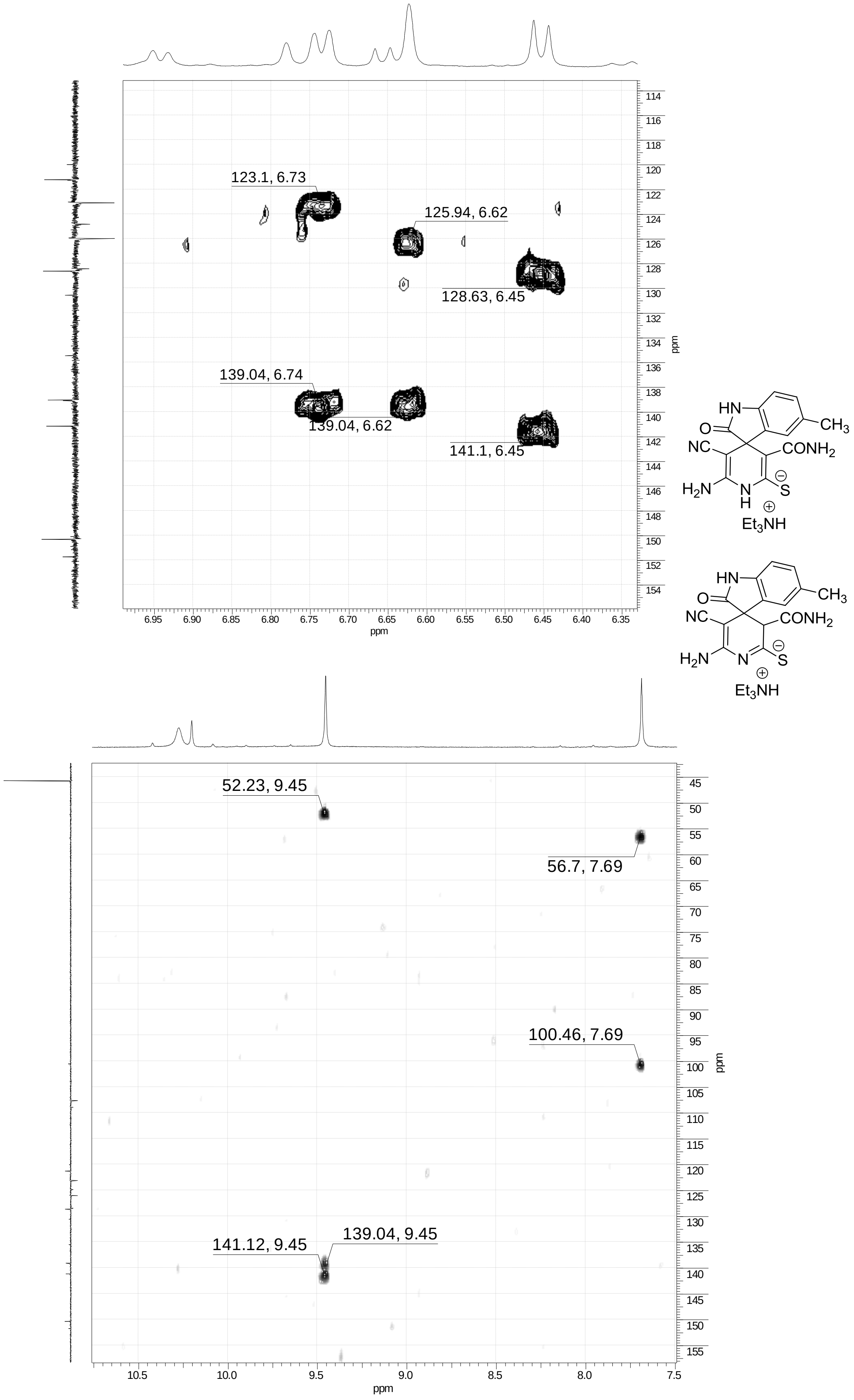

Table S2. The observed correlations in the ^{1}H-^{13}C HSQC and ^{1}H-^{13}C HMBC 2D NMR spectra of thiolate 14a (major 1'H-isomer)

^{13}C chemical shifts are given in **red**, ^{1}H shifts – in **blue**

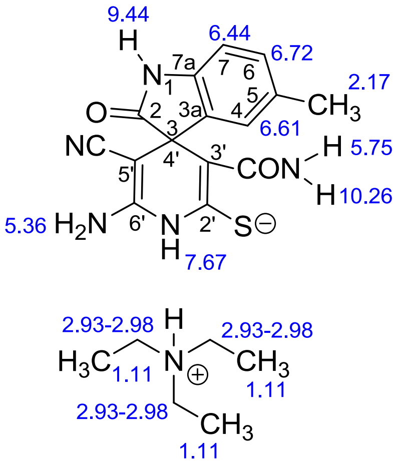

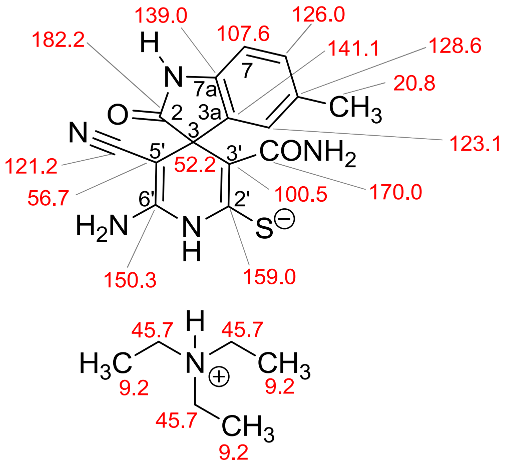

^{1}H NMR shifts, δ, ppm	Correlations in HSQC spectrum, δ, ppm	Correlations in HMBC spectrum, δ, ppm
1.11 (t, ^{3}J = 7.2 Hz, 9H, 3 $C\underline{H}_3CH_2N$)	9.2* ($NCH_2\underline{C}H_3$)	45.7 ($N\underline{C}H_2CH_3$)
2.17 (s, 3H, $Ar\underline{C}H_3$)	20.8* ($ArCH_3$)	123.1* (CH-4 indole), 126.0* (CH-6 indole), 128.6 (C-5 indole)
2.93–2.98 (m, 6H, 3 $C\underline{H}_3CH_2N$)	45.7 ($N\underline{C}H_2CH_3$)	9.2* ($NCH_2\underline{C}H_3$), 45.7 ($N\underline{C}H_2CH_3$)
5.36 (br s, 2H, NH_2)	–	56.7 (C-5')
5.75 (br s, 1H, $C(O)NH_2$)	–	–
6.44 (d, ^{3}J = 7.5 Hz, 1H, H-7 indole)	107.6* (CH-7 indole)	128.6 (C-5 indole), 141.1 (C-3a indole)
6.61 (br s, 1H, H-4 indole)	123.1* (CH-4 indole)	20.8* ($ArCH_3$), 52.2 (C spiro), 126.0* (CH-6 indole), 139.0 (C-7a indole)
6.72 (d, ^{3}J = 7.5 Hz, 1H, H-6 indole)	126.0* (CH-6 indole)	20.8* ($ArCH_3$), 123.1* (CH-4 indole), 139.0 (C-3a indole)
7.67 (s, 1H, NH pyridine)	–	56.7 (C-5'), 100.5 (C-3')
9.44 (s, 1H, NH indole)	–	52.2 (C spiro), 139.0 (C-3a indole), 141.1 (C-7a indole)
10.26 (br s, 1H, $C(O)NH_2$)	–	–

*Signals with a negative phase.

Figure S16. FTIR spectrum of 6'-amino-5'-cyano-5-methyl-2-oxo-2'-thioxo-1,2,2',3'-tetrahydro-1'H-spiro[indole-3,4'-pyridine]-3'-carboxamide 16

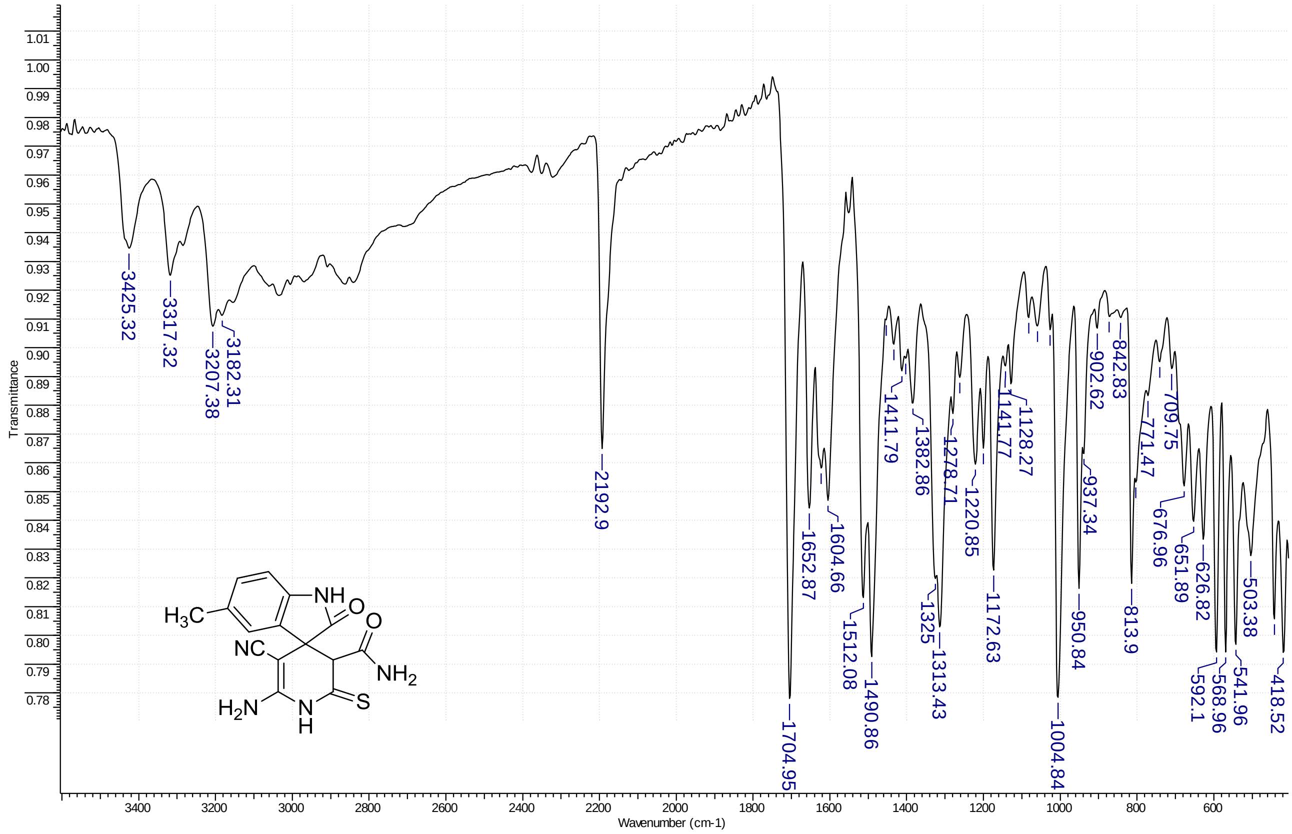

Figure S17. ^{1}H NMR spectrum of 6'-amino-5'-cyano-5-methyl-2-oxo-2'-thioxo-1,2,2',3'-tetrahydro-1'H-spiro[indole-3,4'-pyridine]-3'-carboxamide 16, DMSO-d_6 (400 MHz)

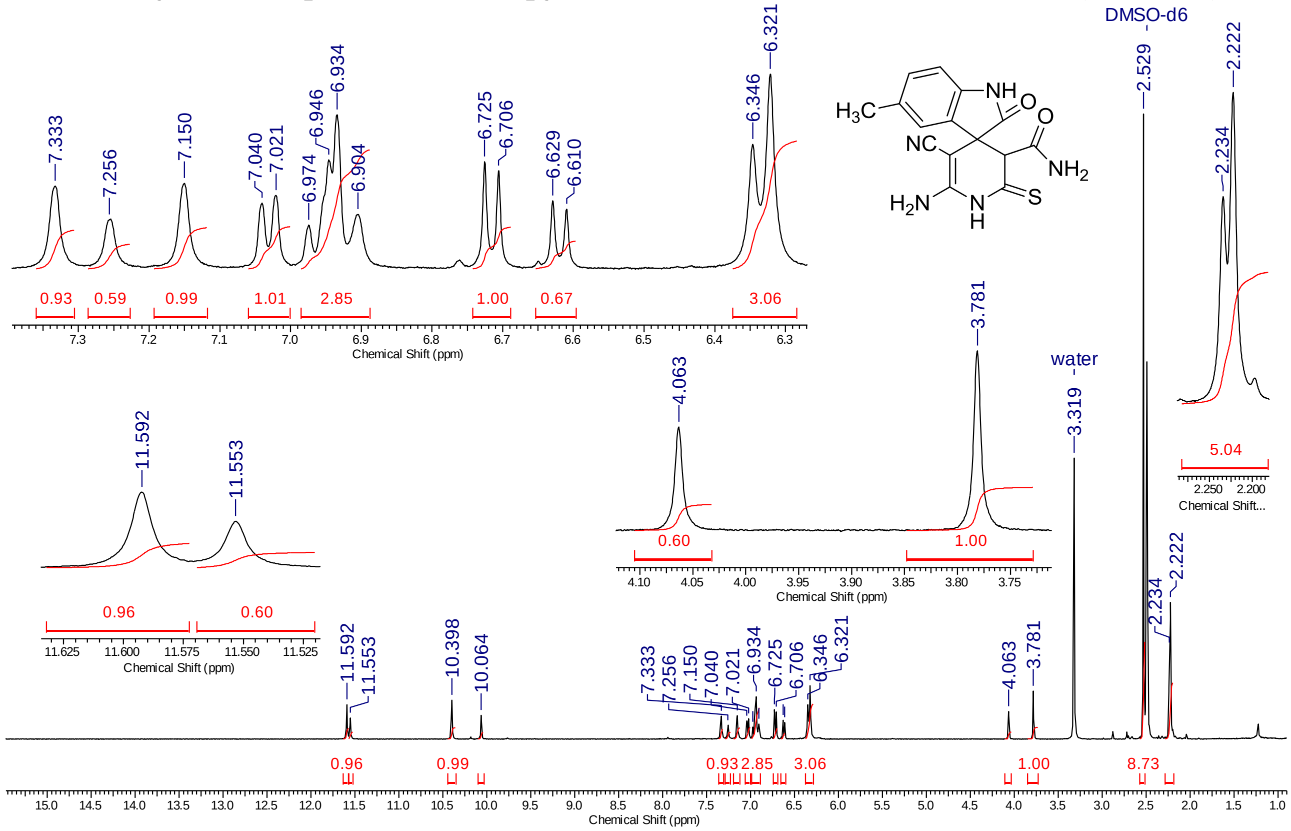

Figure S18. ^{13}C DEPTQ NMR spectrum of 6'-amino-5'-cyano-5-methyl-2-oxo-2'-thioxo-1,2,2',3'-tetrahydro-1'H-spiro[indole-3,4'-pyridine]-3'-carboxamide 16, DMSO-d$_6$ (101 MHz)

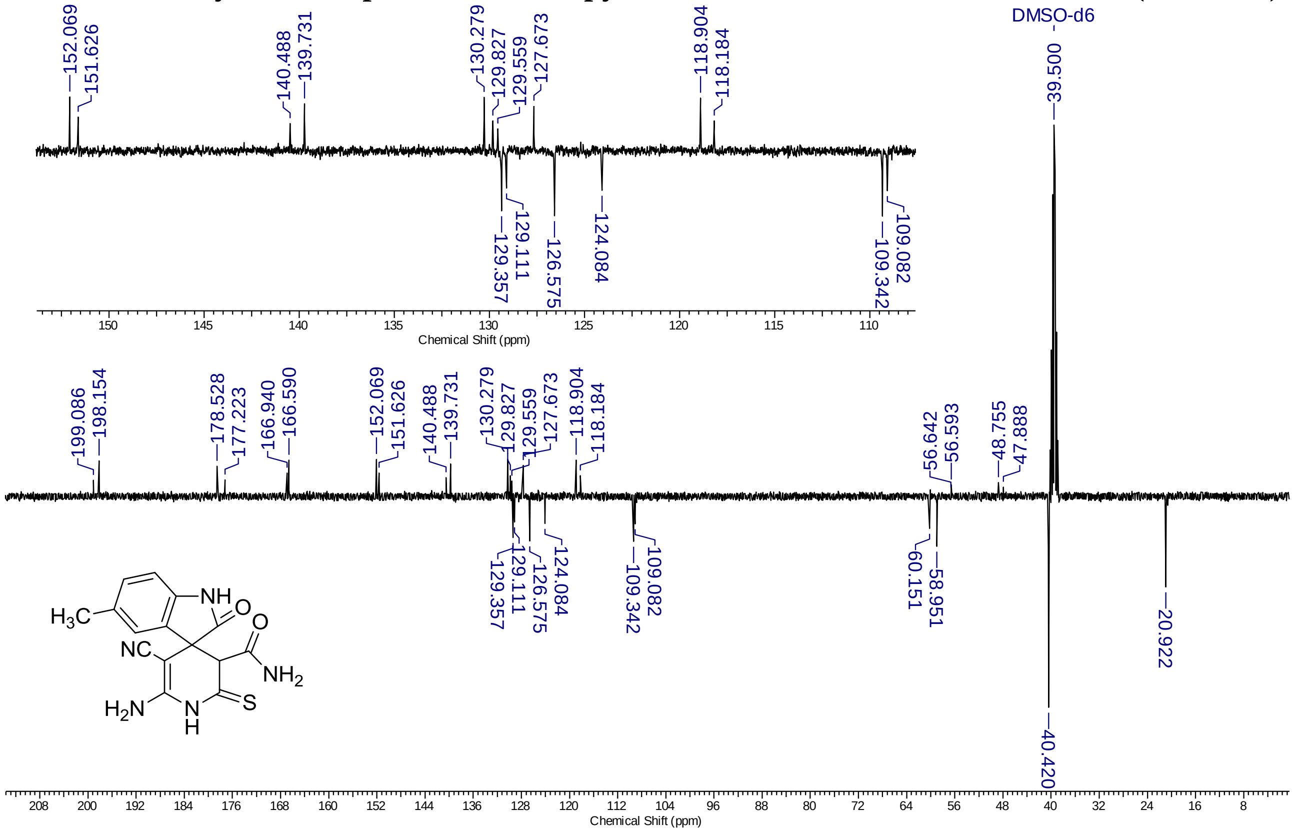

Figure S19. FTIR spectrum of compound 17a

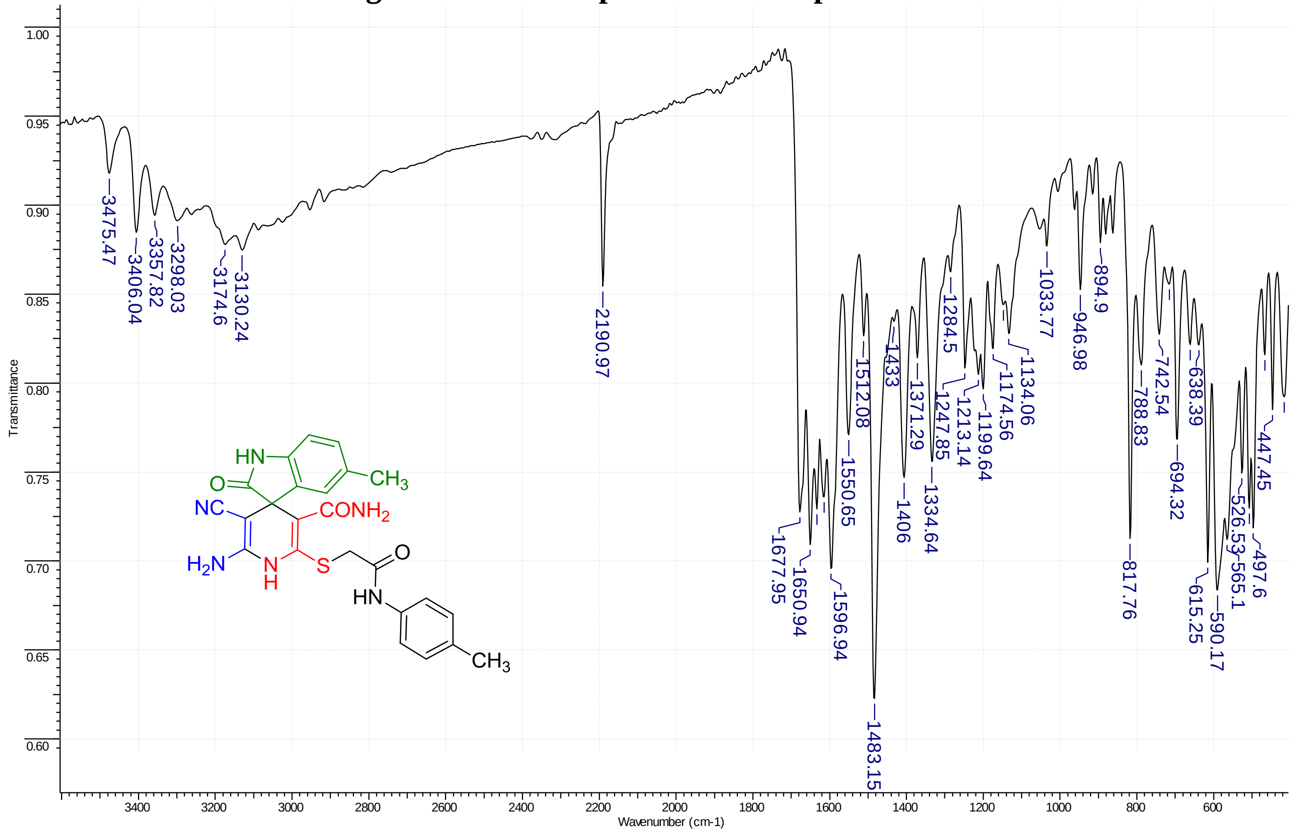

Figure S20. ^{1}H NMR spectrum of compound 17a, DMSO-d_6 (400 MHz)

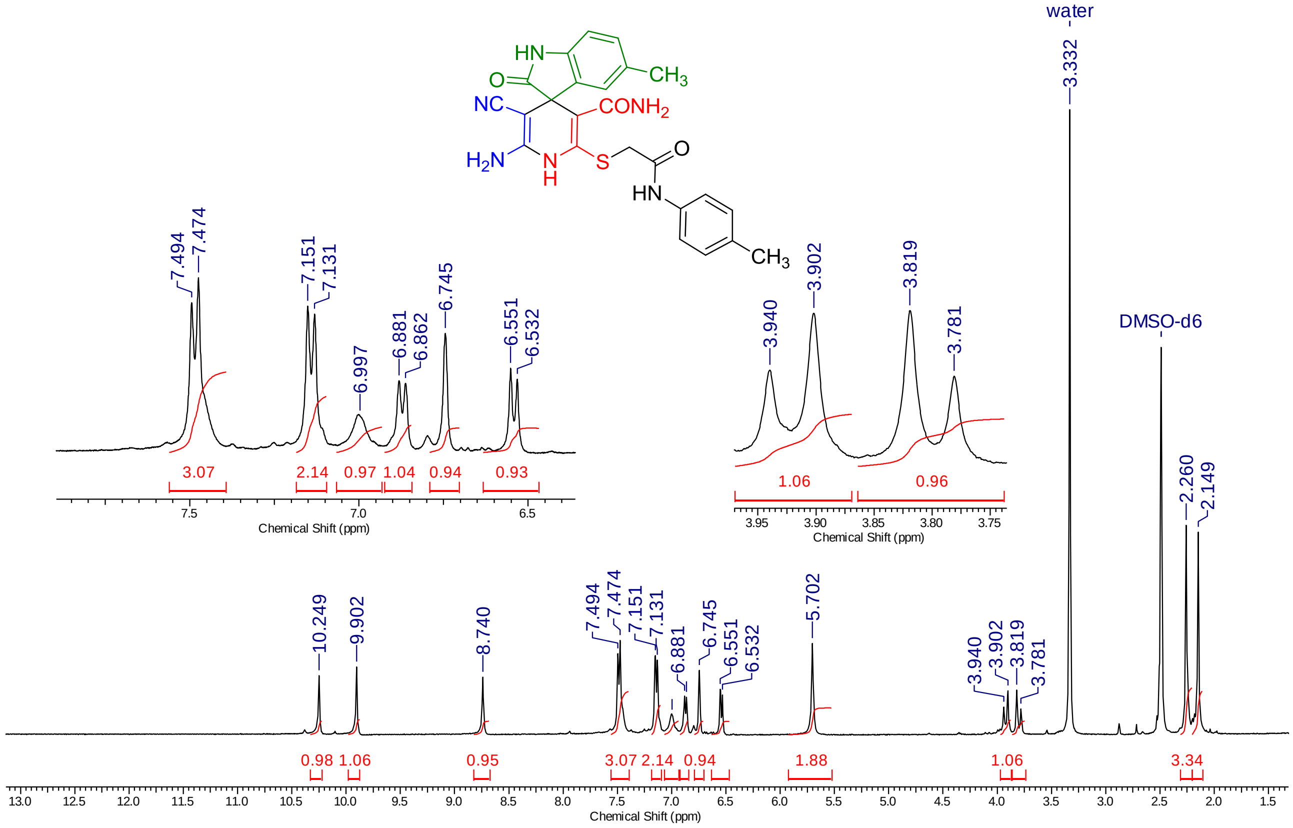

Figure S21. ^{13}C DEPTQ NMR spectrum of compound 17a, DMSO-d_6 (101 MHz)

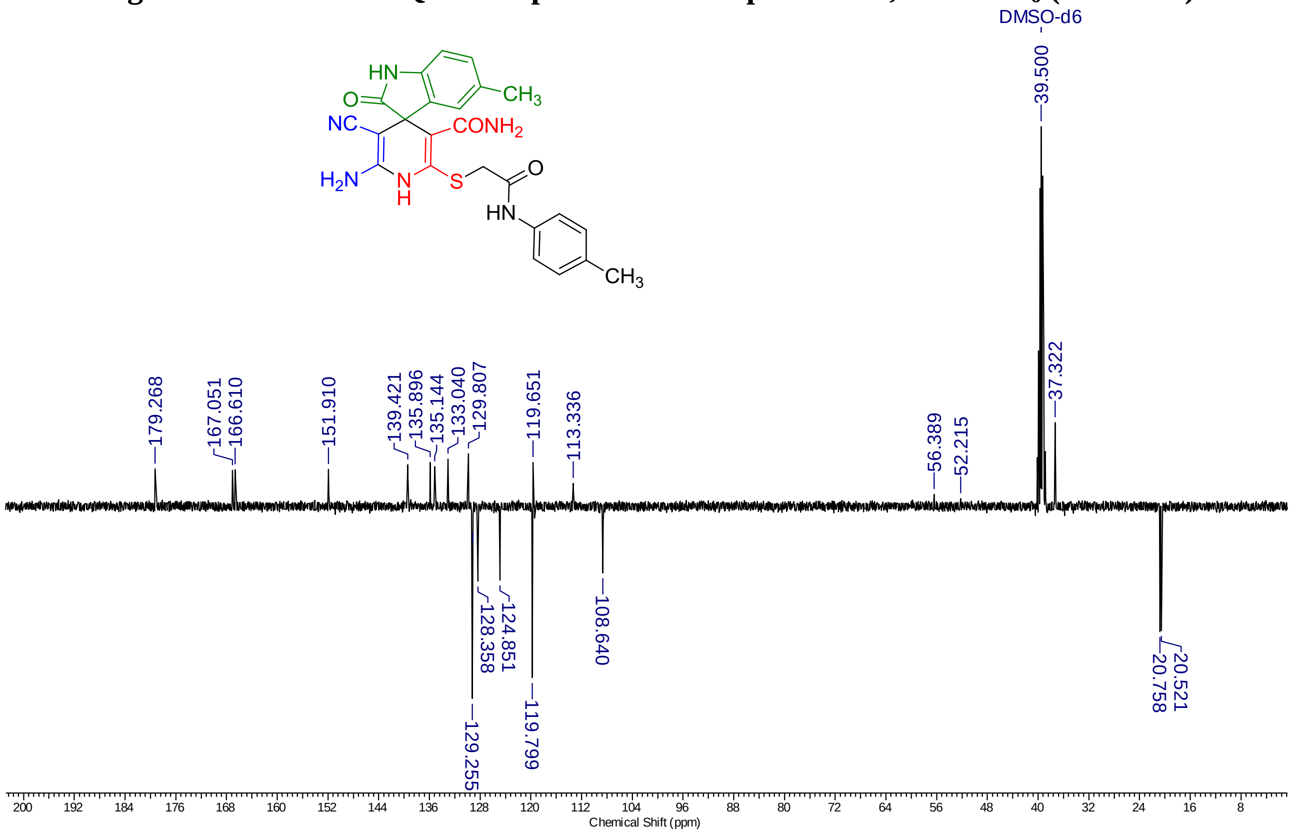

Figure S22. FTIR spectrum of compound 17b

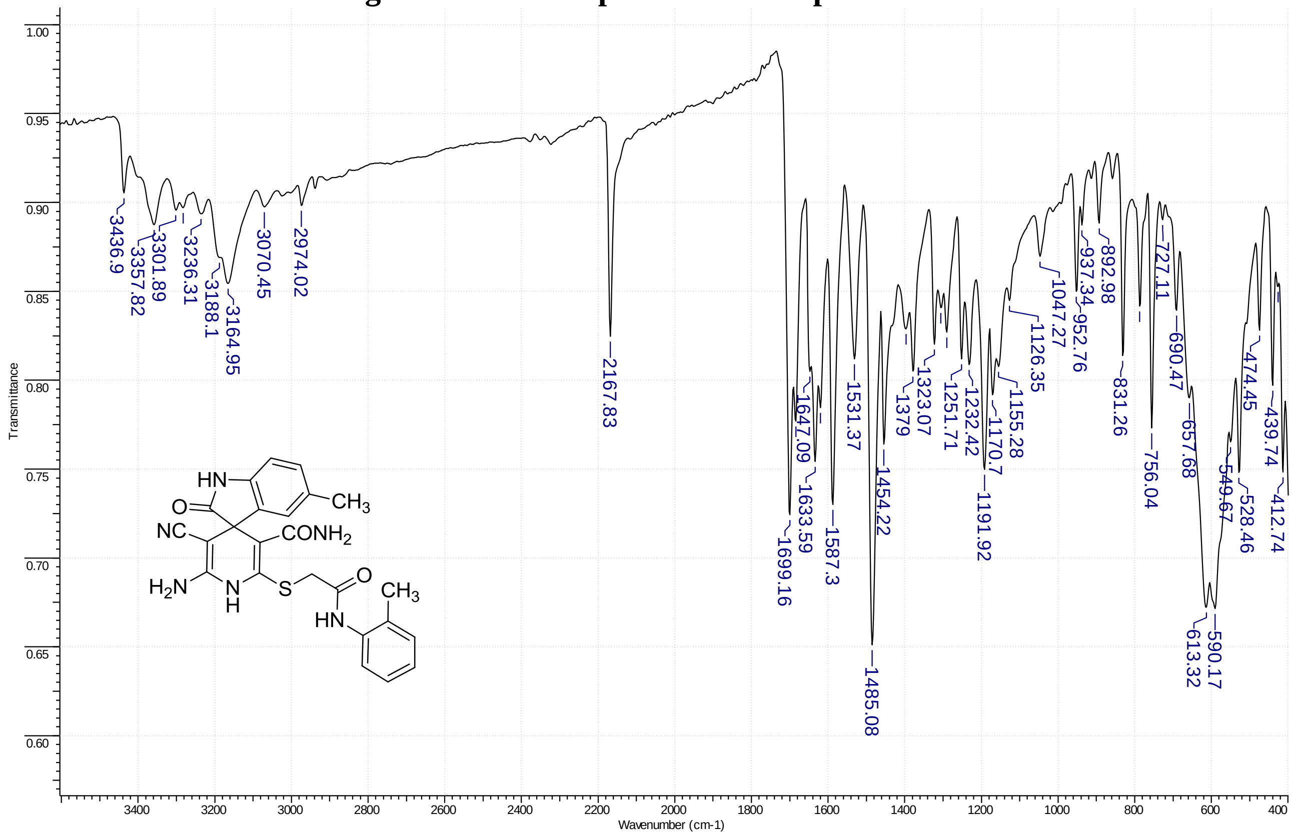

Figure S23. ^{1}H NMR spectrum of compound 17b, DMSO-d$_6$ (400 MHz)

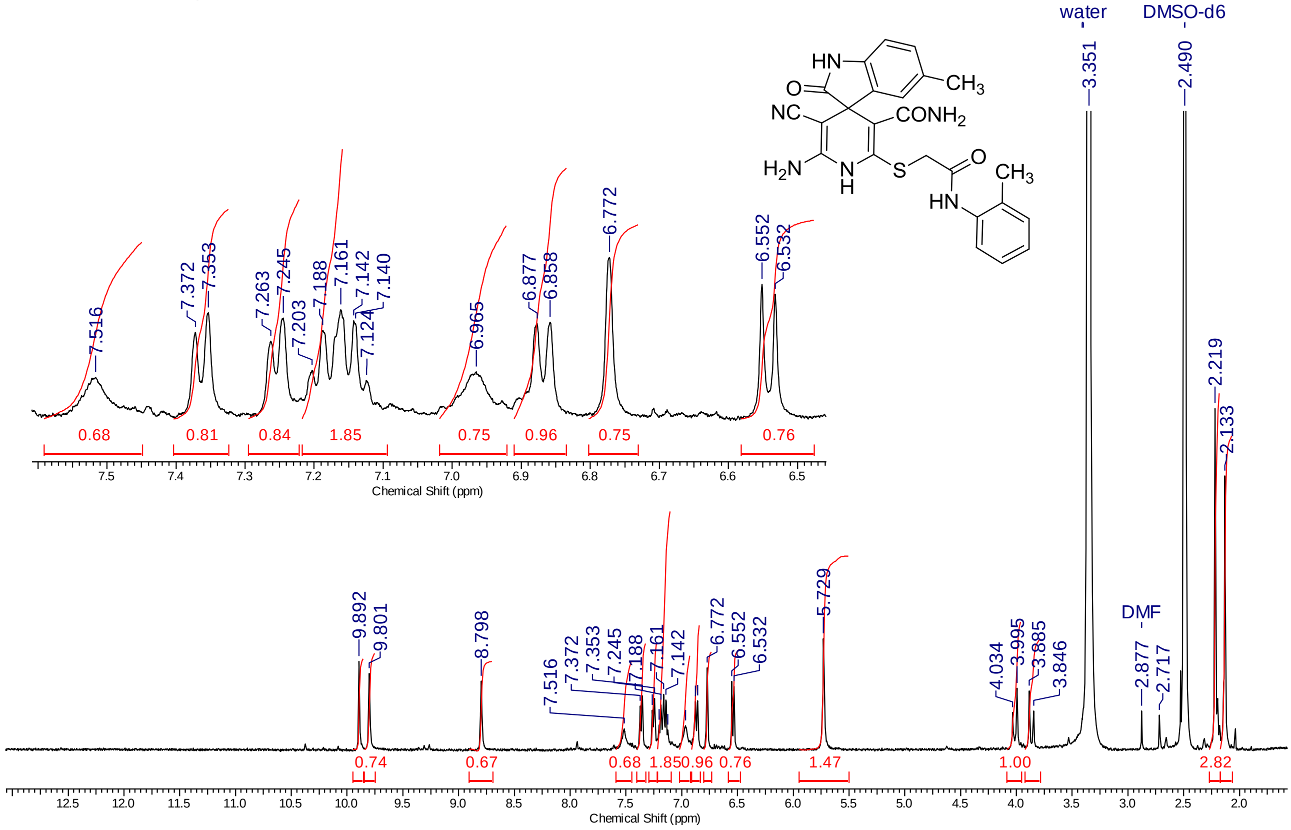

Figure S24. ^{13}**C DEPTQ NMR spectrum of compound 17b, DMSO-d$_6$ (101 MHz)**

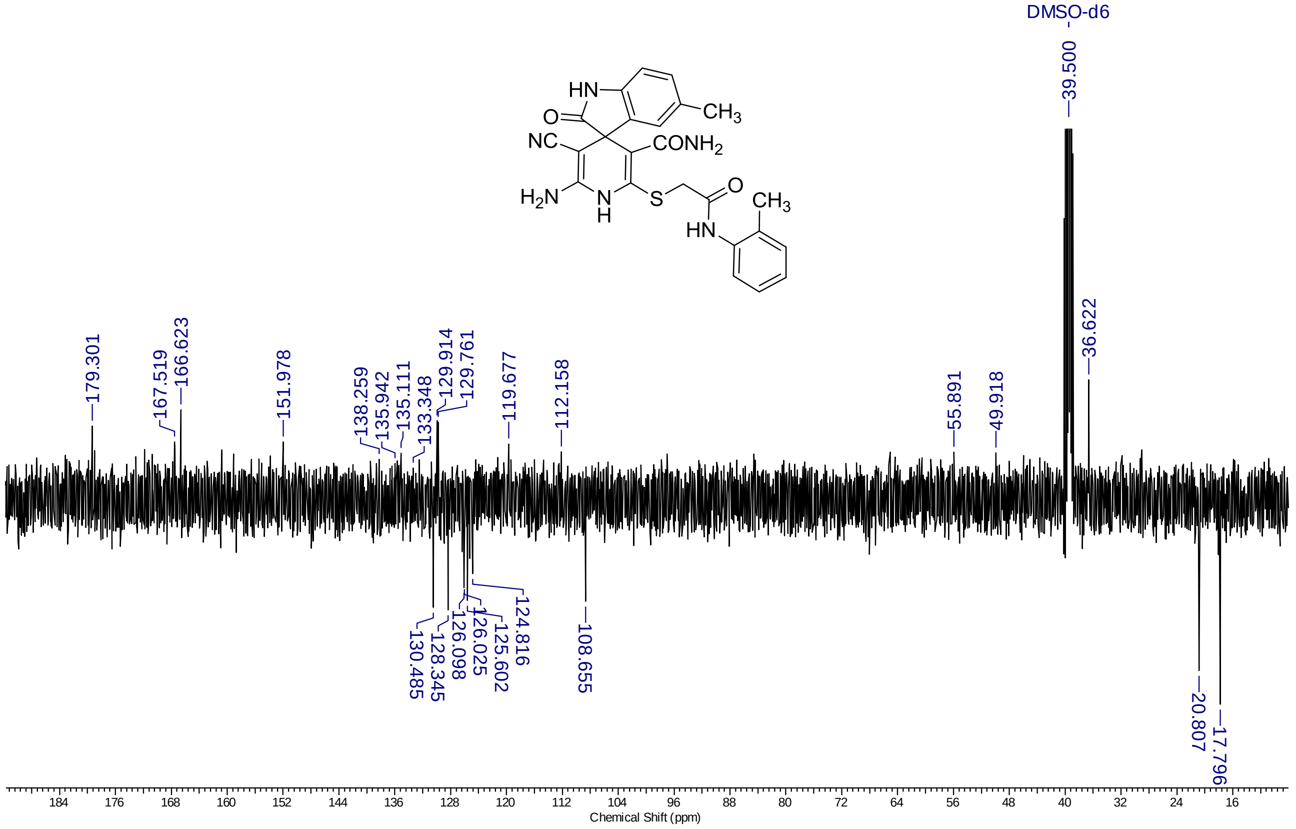

Figure S25. FTIR spectrum of compound 17c+17c-cycl

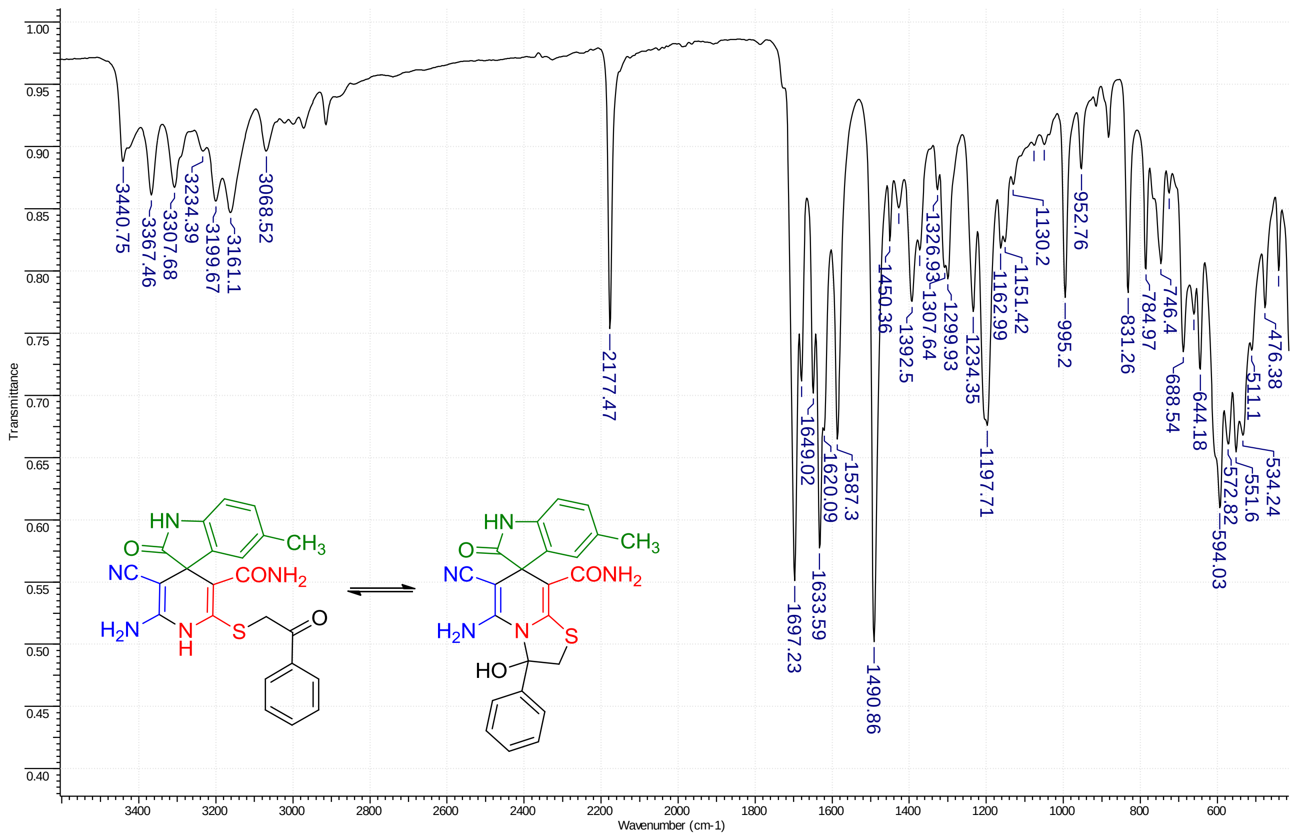

Figure S26. ^{1}**H NMR spectrum of compound 17c+17c-cycl, DMSO-d$_6$ (400 MHz)**

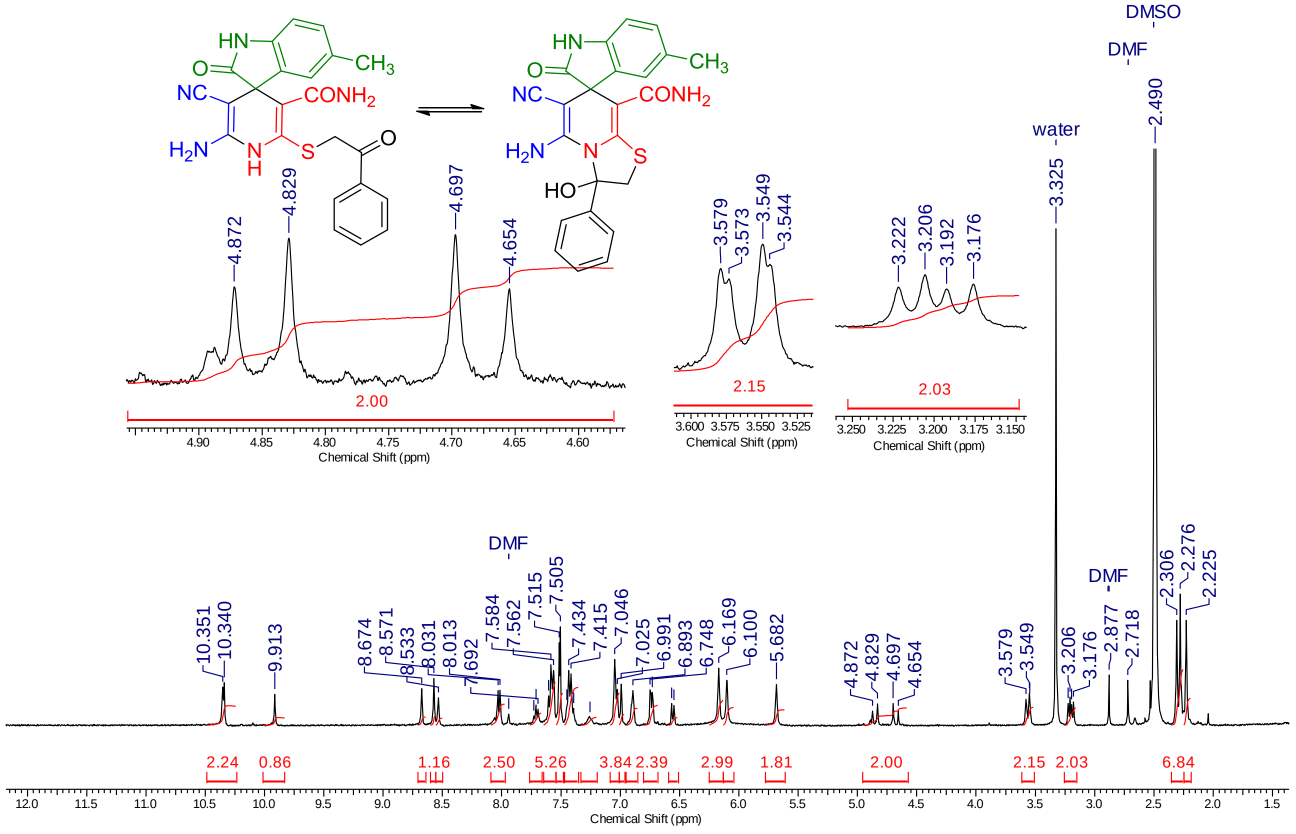

Figure S27. ^{1}**H NMR spectrum of compound 17c+17c-cycl, DMSO-d$_6$ (400 MHz) (*fragment*)**

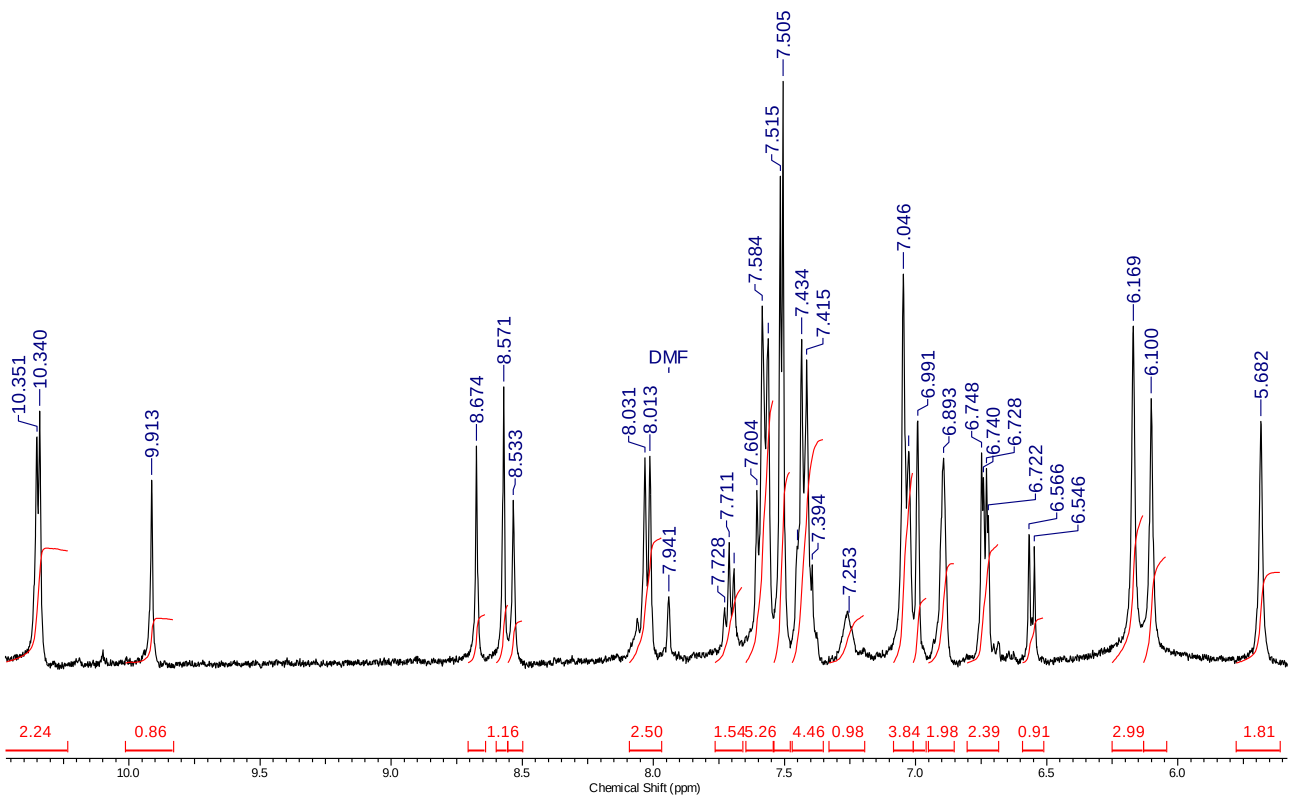

Figure S28. ^{13}C DEPTQ NMR spectrum of compound 17c+17c-cycl, DMSO-d_6 (101 MHz)

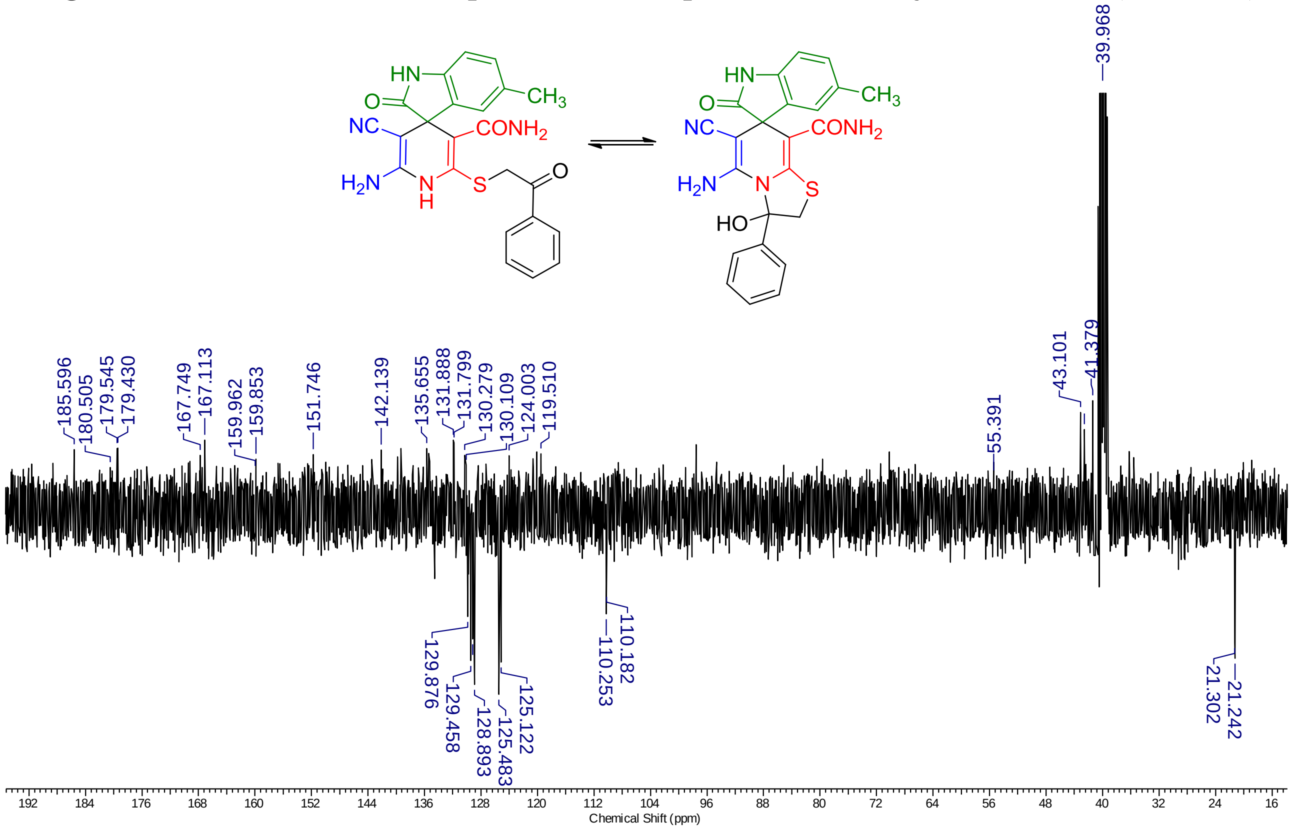

Figure S29. FTIR spectrum of compound 17d+17d-cycl

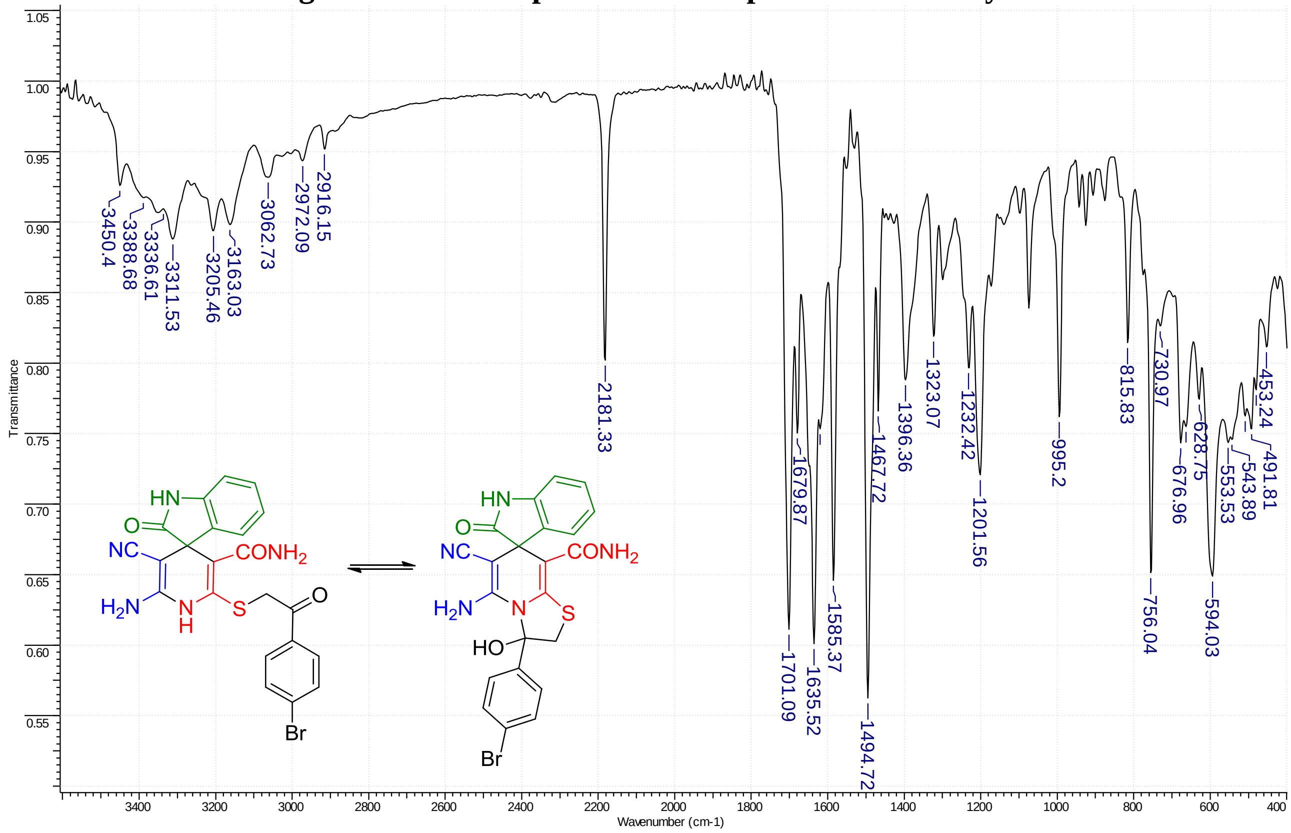

Figure S30. [1]**H NMR spectrum of compound 17d+17d-cycl, DMSO-d$_6$ (400 MHz)**

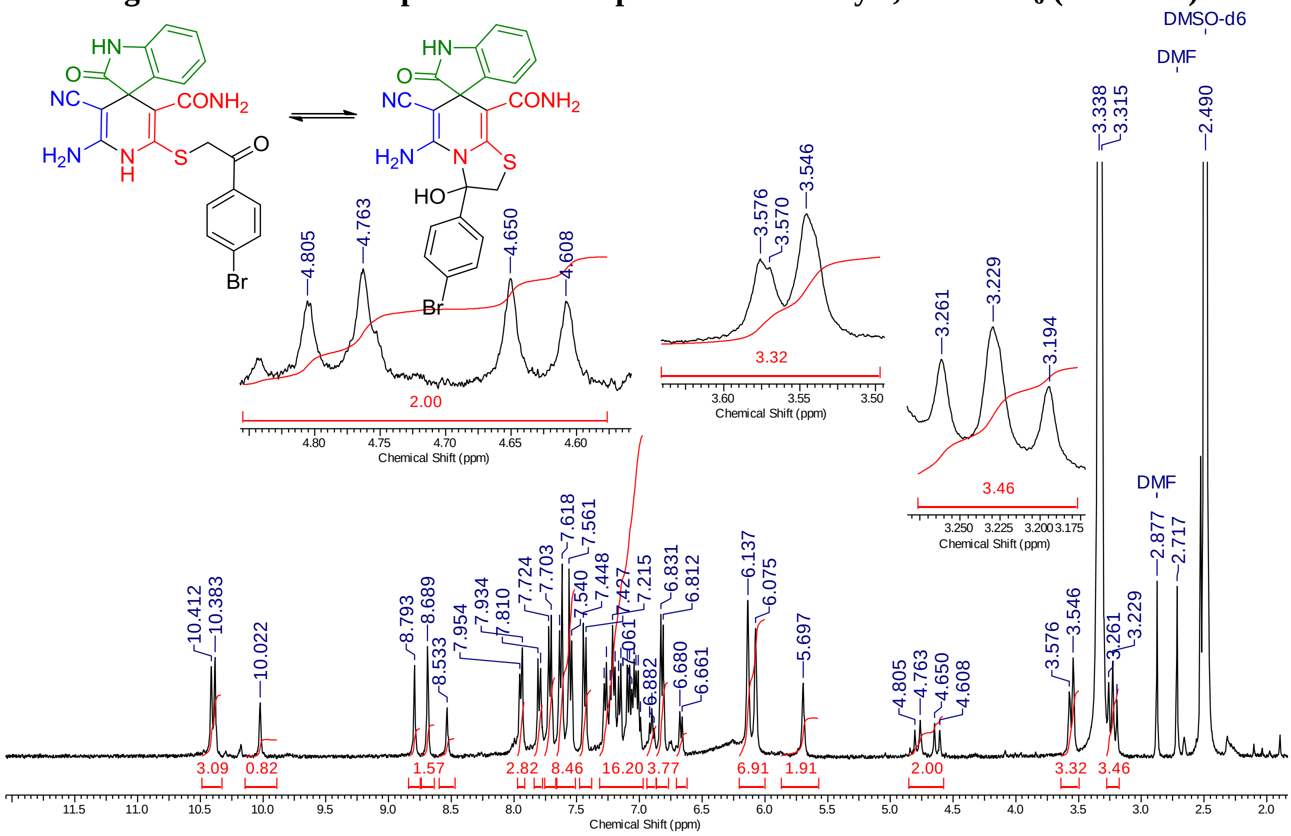

Figure S31. [1]**H NMR spectrum of compound 17d+17d-cycl, DMSO-d$_6$ (400 MHz) (*fragment*)**

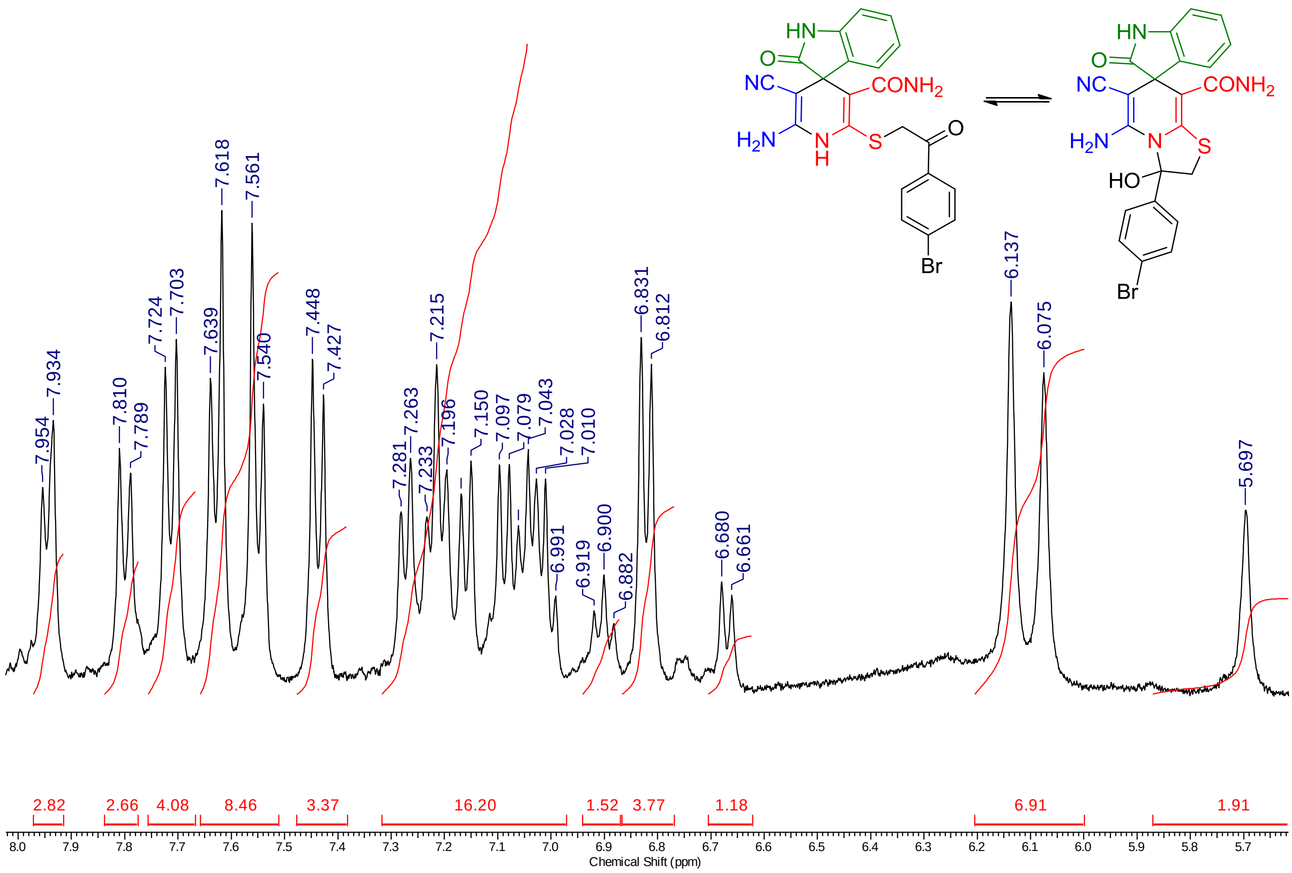

Figure S32. ^{13}C DEPTQ NMR spectrum of compound 17d+17d-cycl, DMSO-d_6 (101 MHz)

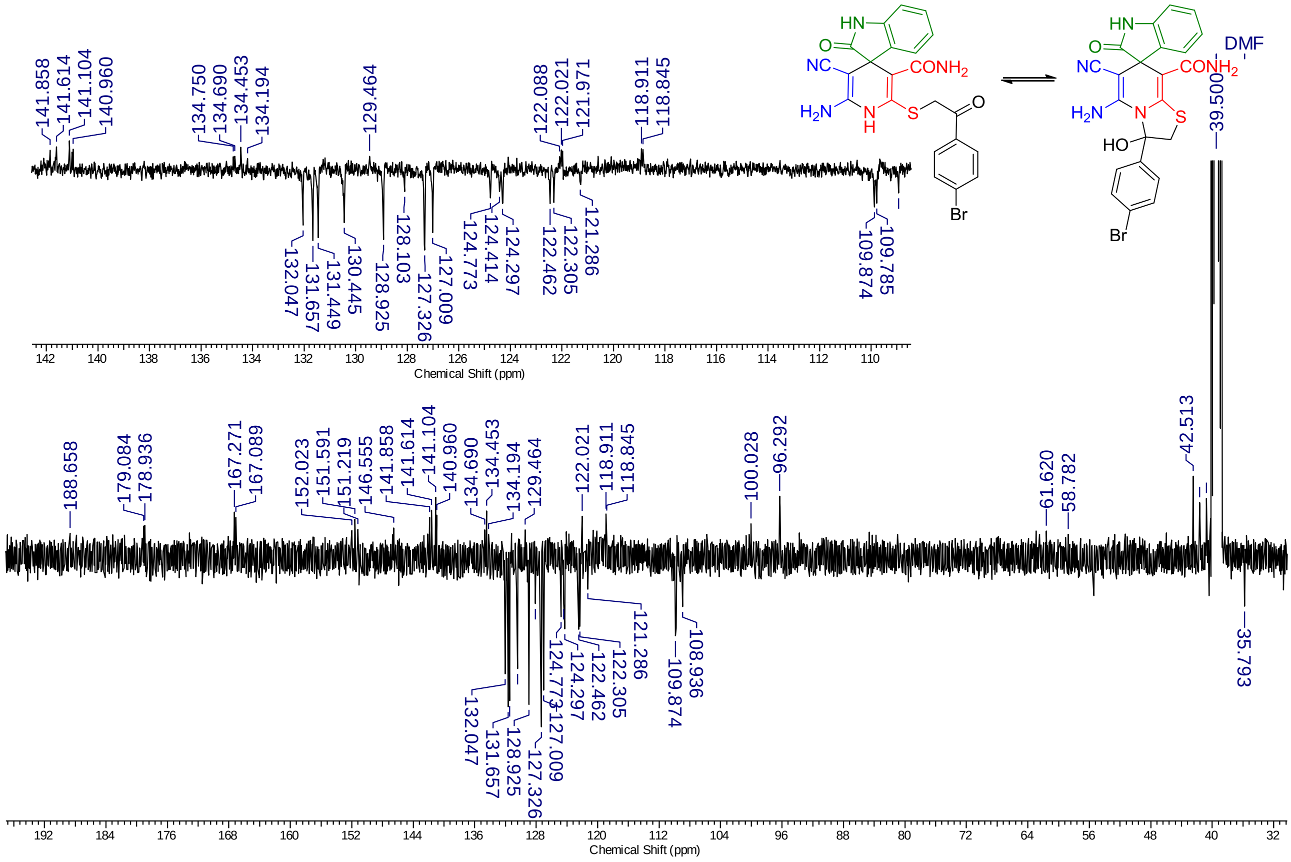

Figure S33. HRMS (ESI) spectrum of thiolates 13a+13b

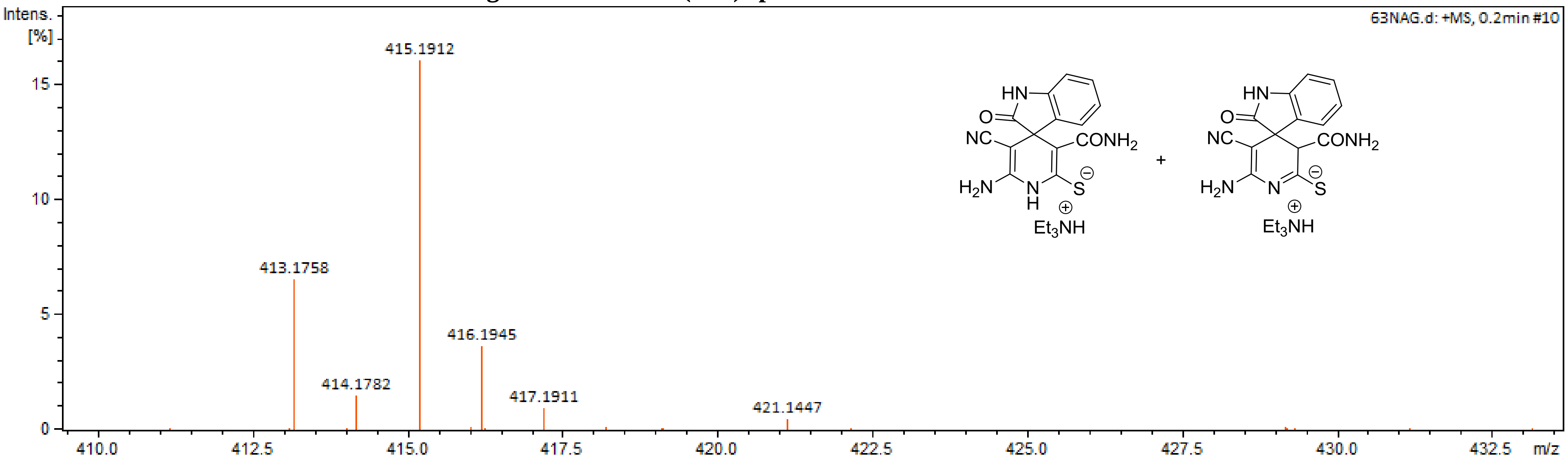

Figure S34. HRMS spectrum of thiolates 14a+14b

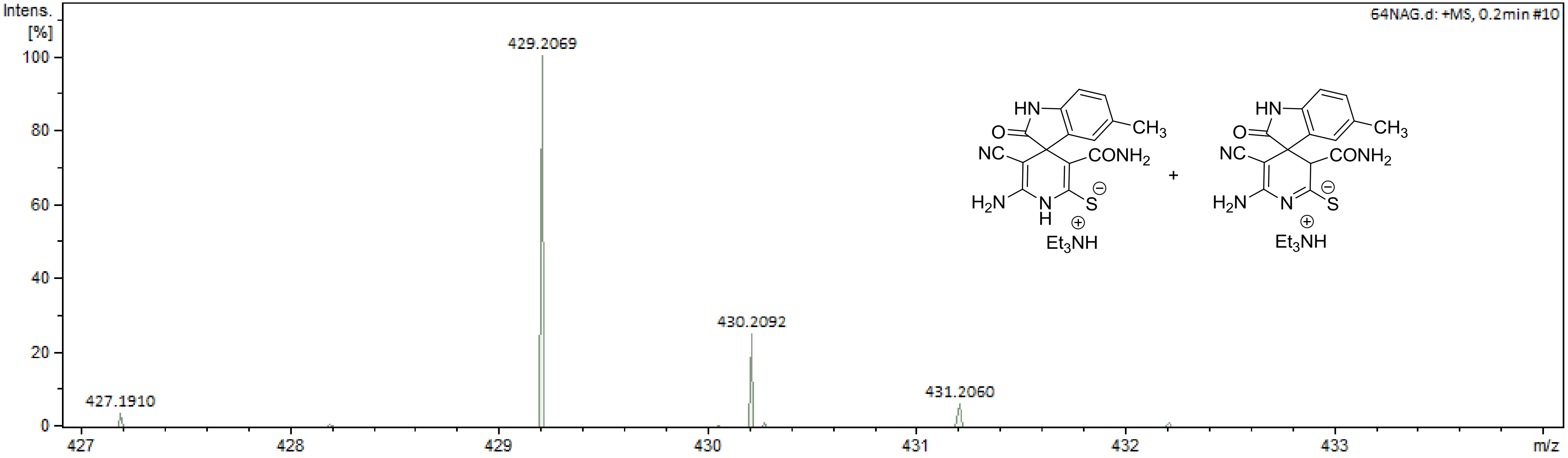

Figure S35. HRMS spectrum of 6'-amino-5'-cyano-5-methyl-2-oxo-2'-thioxo-1,2,2',3'-tetrahydro-1'H- spiro[indole-3,4'-pyridine]-3'-carboxamide 16

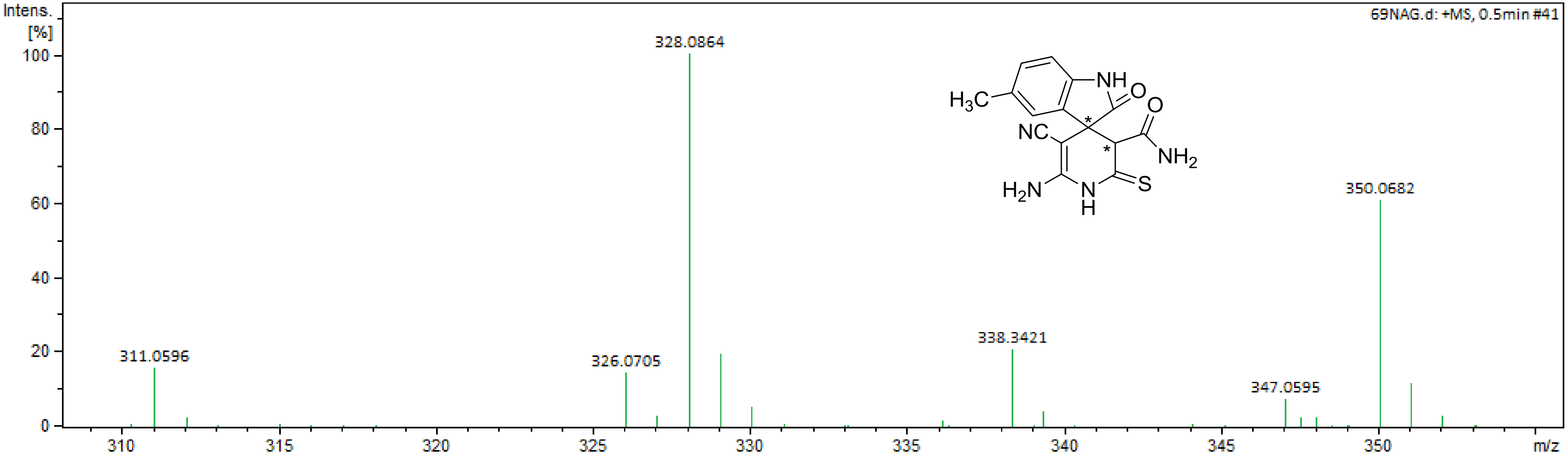

Figure S36. HRMS spectrum of compound 17a

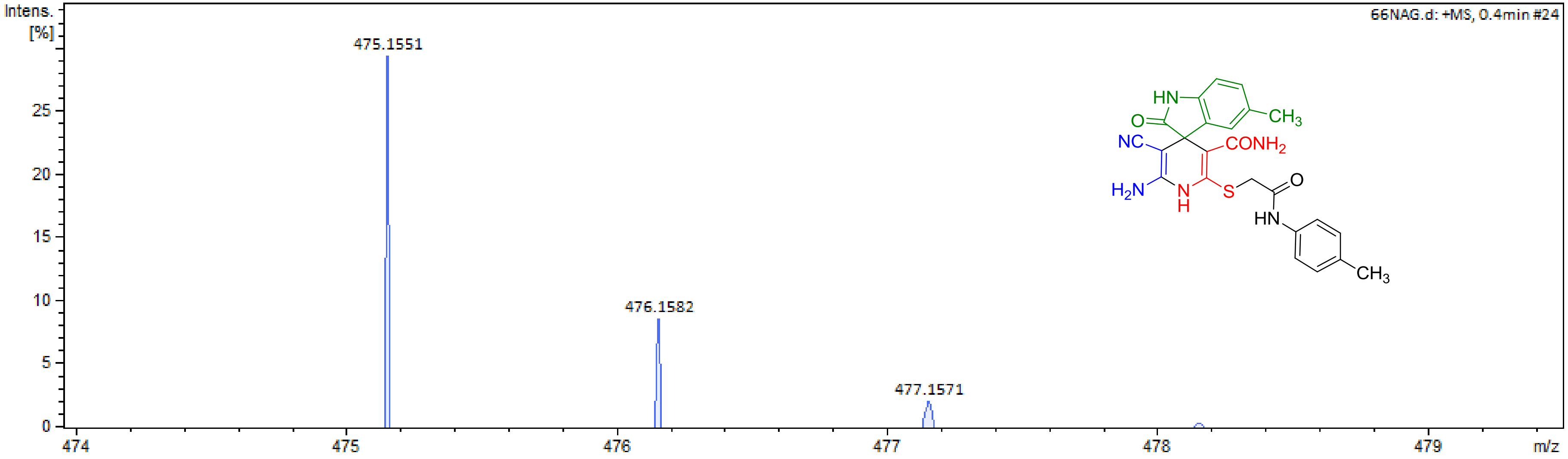

Figure S37. HRMS spectrum of compound 17b

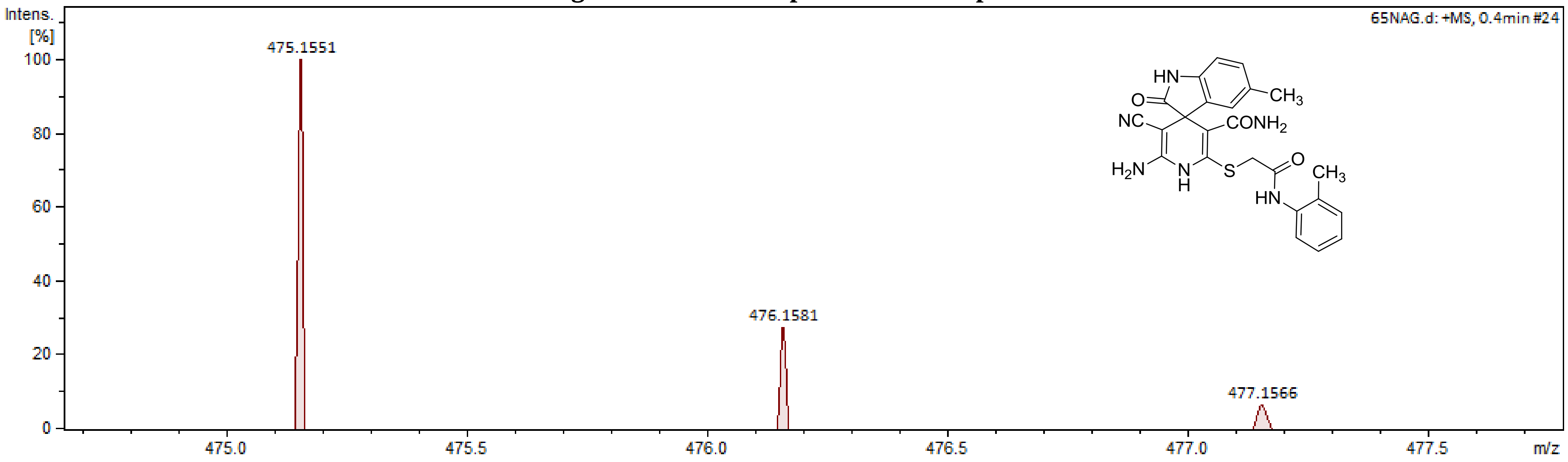

Figure S38. HRMS spectrum of compound 17c+17c-cycl

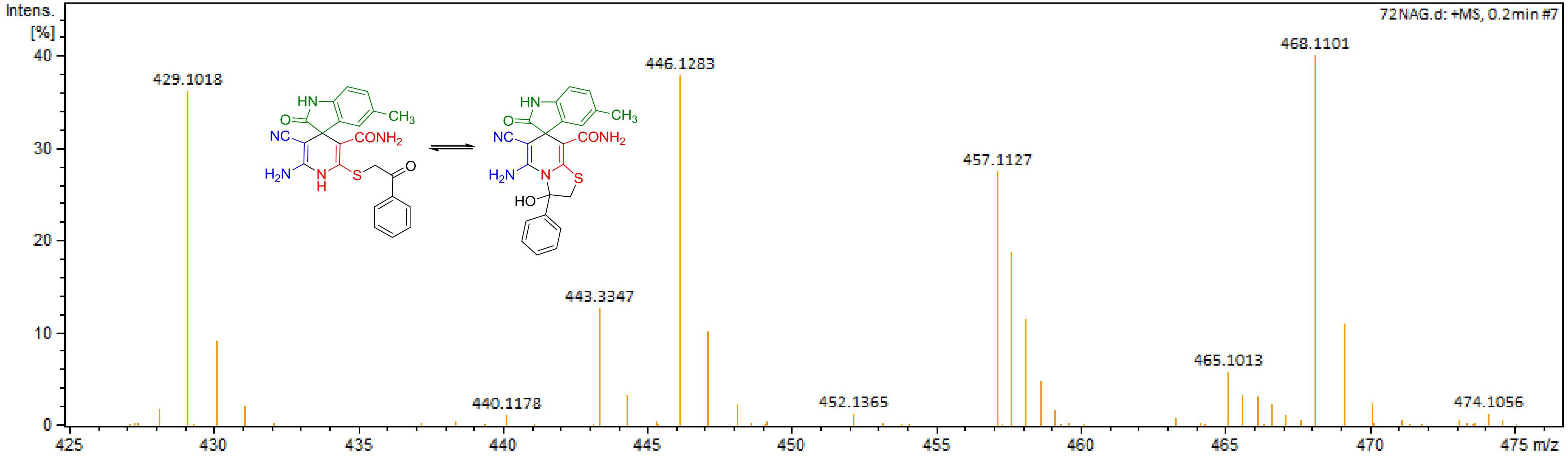

Figure S39. HRMS spectrum of compound 17d+17d-cycl

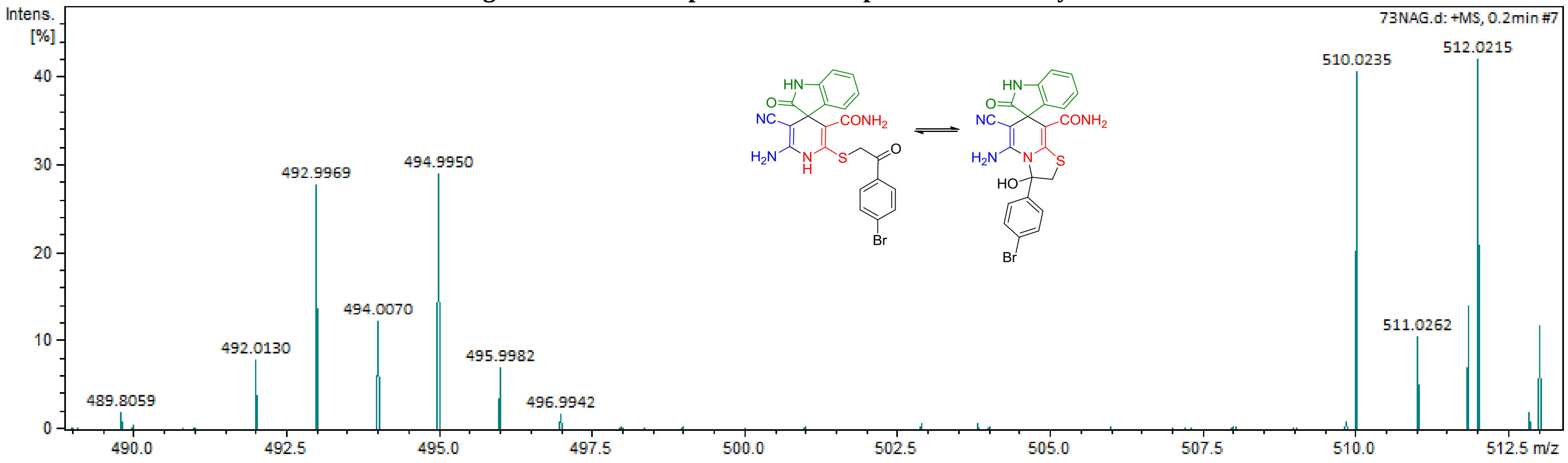

Supplement: Supplementary file 1 [file molecules-28-03161-s001.zip › molecules-2280714-supplementary.pdf]
